# Hybrid Metasurfaces for Perfect Transmission and Customized Manipulation of Sound Across Water–Air Interface

**DOI:** 10.1002/advs.202207181

**Published:** 2023-04-20

**Authors:** Hong‐Tao Zhou, Shao‐Cong Zhang, Tong Zhu, Yu‐Ze Tian, Yan‐Feng Wang, Yue‐Sheng Wang

**Affiliations:** ^1^ Department of Mechanics School of Mechanical Engineering Tianjin University Tianjin 300350 China; ^2^ Institute of Engineering Mechanics Beijing Jiaotong University Beijing 100044 China

**Keywords:** acoustic metasurfaces, cross‐media manipulation, impedance matching, topology optimization, water–air interface

## Abstract

Extreme impedance mismatch causes sound insulation at water–air interfaces, limiting numerous cross‐media applications such as ocean‐air wireless acoustic communication. Although quarter‐wave impedance transformers can improve transmission, they are not readily available for acoustics and are restricted by the fixed phase shift at full transmission. Here, this limitation is broken through impedance‐matched hybrid metasurfaces assisted by topology optimization. Sound transmission enhancement and phase modulation across the water–air interface are achieved independently. Compared to the bare water–air interface, it is experimentally observed that the average transmitted amplitude through an impedance‐matched metasurface at the peak frequency is enhanced by ≈25.9 dB, close to the limit of the perfect transmission 30 dB. And nearly 42 dB amplitude enhancement is measured by the hybrid metasurfaces with axial focusing function. Various customized vortex beams are experimentally realized to promote applications in ocean‐air communication. The physical mechanisms of sound transmission enhancement for broadband and wide‐angle incidences are revealed. The proposed concept has potential applications in efficient transmission and free communication across dissimilar media.

## Graphical abstract

Hybrid metasurfaces are proposed to realize perfect transmission and customized manipulation of sound across the water–air interface. Exploiting the fluid–solid interactions, an inverse‐design strategy based on topology optimization is developed to design the impedance‐matched hybrid metasurfaces. Various wavefront manipulations are demonstrated in simulations and experiments. The presented approach will facilitate potential applications of metasurfaces in cross‐media acoustic communication.

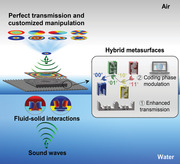

## 1 Introduction

Cross‐media transmission modulation of waves is receiving considerable attention^[^ ^1^, ^2^, ^3^, ^4^, ^5^, ^6^ ^]^ due to its broad application prospects in many engineering fields such as wireless communication,^[^ ^7^, ^8^ ^]^ energy transfer,^[^ ^9^ ^]^ detection,^[^ ^10^, ^11^ ^]^ and imaging.^[^ ^12^, ^13^ ^]^ Full manipulation of the wave across dissimilar media means that both the amplitude and phase of transmitted waves are controllable.^[^ ^14^, ^15^ ^]^ However, the extremely low transmission across the interface due to impedance mismatch between dissimilar media first poses a huge challenge on the modulation of amplitude. One of the most common examples is sound transmission through the water–air interface.^[^ ^16^ ^]^ Due to the sharp contrast in acoustic impedance (≈3600 times), only 0.1% of the acoustic energy can be naturally transmitted between water and air.^[^ ^17^ ^]^ Whereas, sound waves are by far the only practical means of underwater wireless communication because of their low attenuation compared to electromagnetic waves.^[^ ^18^, ^19^, ^20^ ^]^ If an efficient water‐to‐air sound transmission is realized, the ocean covering 70% of Earth's surface and atmosphere will be effectively connected by sound waves.^[^ ^21^ ^]^ Light‐weight aerial loudspeakers can be immersed underwater to efficiently emit sound waves, and high‐sensitivity microphones can also be exploited to achieve high signal‐to‐noise ratio detection.^[^ ^22^ ^]^ The realization of cross‐media extreme transmission and manipulation will significantly improve the performance of underwater acoustic devices and facilitate wireless underwater to air communications.^[^ ^21^, ^22^ ^]^

To enhance sound transmission through the water–air interface, several efforts have been made during the past few years. Rigorous theoretical derivations suggest that acoustic energy carried by spatially decaying waves, such as inhomogeneous or evanescent plane waves, can be effectively transmitted across the interface.^[^ ^23^, ^24^ ^]^ This is mainly attributed to that the decaying component introduced to the incident wave results in a nonzero propagating wave component in the transmitted fields.^[^ ^23^, ^24^ ^]^ In addition, it is theoretically and numerically demonstrated that tunable sound transmission at an impedance‐mismatched fluidic interface can be achieved by dynamically adjusting the impedance characteristics of a composite waveguide.^[^ ^25^ ^]^ Metasurfaces,^[^ ^26^, ^27^, ^28^, ^29^, ^30^ ^]^ which have been used to achieve high‐efficiency reflection,^[^ ^31^, ^32^ ^]^ transmission,^[^ ^33^, ^34^, ^35^, ^36^ ^]^ absorption,^[^ ^37^, ^38^ ^]^ and isolation^[^ ^39^, ^40^ ^]^ in the homogeneous background medium, were also applied to solve the extreme mismatch at the water–air interface.^[^ ^21^, ^22^, ^41^, ^42^ ^]^ By employing a loaded membrane‐type metasurface, it is experimentally observed that nearly 30% of incident acoustic power at around 700~Hz was transferred from water to air in a tube.^[^ ^22^ ^]^ Based on a mechanically‐rigid metasurface composed of resonance‐matching and labyrinthine‐shaped components, sound intensity enhancement exceeding 38 dB is experimentally measured at 8 kHz by combining a focusing phase distribution, realizing the remote water‐to‐air eavesdropping with a large signal‐to‐noise ratio.^[^ ^41^ ^]^ Besides, metasurfaces made of coupled resonant bubbles immersed underwater are proven to be another effective way to improve the sound transmission at the interface.^[^ ^21^, ^42^, ^43^, ^44^, ^45^ ^]^ The underlying mechanisms of high‐efficiency transmissions are described by the mass‐spring resonance models.^[^ ^21^, ^44^ ^]^ However, due to the strict dependence of resonant frequency on the bubble size and immersion depth, the operating frequency of bubble‐type metasurfaces is generally restricted below 4 kHz in the existing experiments.^[^ ^21^ ^]^ For the enhanced transmission at intermediate frequencies around 10 kHz, an artificial lotus metasurface composed of a superhydrophobic aluminum plate is proposed based on a similar principle.^[^ ^42^ ^]^ The immersing part of the superhydrophobic aluminum plate in water can form µm‐scale air layers to make up the mass‐spring resonance system.^[^ ^42^ ^]^ But the necessary hydrophobic treatment on metal materials inevitably brings some tedious operations to large‐scale fabrication and preparation of samples.^[^ ^42^ ^]^ In general, alternative simple and practical approaches with the aid of new physical mechanisms remain to be explored.

Actually, the classical quarter‐wavelength theorem^[^ ^46^, ^47^ ^]^ indicates that perfect energy transmission across dissimilar media can be realized when the matched layer has a particular acoustic impedance of $\sqrt{Z_{1}Z_{2}}$ and a certain thickness *d* = λ_*d*_/4, where *Z*_1_, *Z*_2_, and λ_*d*_ are the characteristic acoustic impedances of the two different media and the wavelength in the matched layer, respectively. Hence, it is possible to provide a general solution to solve ultra‐low transmission at the interface.^[^ ^3^, ^48^ ^]^ However, for the water‐to‐air case, such a matched layer with acoustic impedance about 60 times to that for air is hardly available in nature.^[^ ^22^ ^]^ How to explore a viable way to construct the matched layer with extreme material properties is of great significance. Apart from this, previous studies have mainly focused on improving sound transmission between water and air.^[^ ^21^, ^42^, ^43^, ^44^, ^45^ ^]^ Wavefront manipulation of transmitted sound waves across the water–air interface has always been an overlooked but essential issue, which can bring a broad range of remarkable functionalities.^[^ ^41^, ^49^ ^]^ In addition, based on the effective medium approximation, the transmission phase will be locked to ±π/2 for full transmission, disabling the wavefront phase control ability. Therefore, how to achieve phase manipulation of a wave under full transmission is still a rather challenging task.

In this work, we theoretically propose a universal approach to address the challenges of transmission enhancement and phase modulation on sound propagation across the water–air interface. These two functions are well integrated by exploiting hybrid metasurfaces (labeled as ${\mathrm{MS}}_{1}$ and ${\mathrm{MS}}_{2}$) on the decoupled control of transmitted amplitude and phase across the water–air interface. The topology optimization is applied to systematically develop an inverse design method to separately design the hybrid metasurfaces with unitary transmission (${\mathrm{MS}}_{1}$) and desired phase shifts (${\mathrm{MS}}_{2}$). The proposed ${\mathrm{MS}}_{1}$ consists only of a single‐phase solid material. The inversely designed ${\mathrm{MS}}_{2}$ is encoded and then assembled by discrete unit cells with unitary transmission and four desired phase shifts according to the customized phase distribution. Multiple acoustic functions involving axial focusing and vortex beams carrying various orbital angular momentums are implemented to demonstrate the ability of wavefront manipulation under enhanced transmission. Water‐to‐air acoustic experiments are further conducted to validate the performance of hybrid metasurfaces. The underlying physical mechanism of ${\mathrm{MS}}_{1}$ for enhancing water‐to‐air sound transmission is also revealed. The presented coalescence of high‐efficiency transmission and customized wavefront manipulation greatly demonstrates the full‐wave tailoring capabilities of metasurfaces, and paves the way for applications of advanced transmedia devices in wireless communications, medical monitoring, smart sensors, and so on.

## 2 Results

### 2.1 Theory of Achieving Perfect Transmission and Phase Shifting

**Figure 1:**
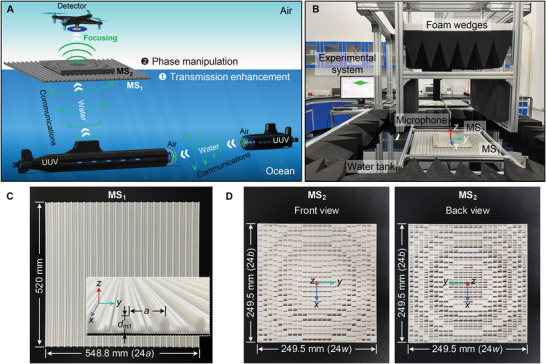
The proposed concept of hybrid metasurfaces for perfect transmission and customized manipulation of sound across the water–air interface. Panel (A) schematically illustrates the sound manipulation by hybrid metasurfaces (${\mathrm{MS}}_{1}$ and ${\mathrm{MS}}_{2}$) across the water–air interface, showing the potential applications in ocean‐air and underwater acoustic wireless communication. Panel (B) shows the experimental setup of hybrid metasurfaces for perfect transmission and customized manipulation between water and air. The sound waves emitted from a transducer under the water tank are transmitted to the air by ${\mathrm{MS}}_{1}$, manipulated by ${\mathrm{MS}}_{2}$, and finally collected by an aerial microphone. The photographs of fabricated hybrid metasurfaces (${\mathrm{MS}}_{1}$ and ${\mathrm{MS}}_{2}$ with axial focusing function as an example) are shown in panels (C) and (D), where the inset in panel (C) shows the cross‐section of the ${\mathrm{MS}}_{1}$ sample.

**Figure** **1**A illustrates the schematic diagram for achieving perfect transmission and customized manipulation by hybrid metasurfaces. ${\mathrm{MS}}_{1}$ floating on the water acts as an impedance‐matched layer between water and air to enhance sound transmission. A digital coding metasurface (${\mathrm{MS}}_{2}$), placed on top of ${\mathrm{MS}}_{1}$, has customized phase distribution to arbitrarily tailor wavefronts without transmission attenuation. In this case, the detector in the air can easily collect sound signals sent by underwater unmanned vehicles (UUV), and bulky underwater acoustic systems can also be avoided in communication between UUVs by exploiting efficient air–water–air sound propagation. To demonstrate the practicability of the proposed concept, a multi‐layer sound transmission model is established based on the effective medium theory.^[^ ^46^ ^]^ Both metasurfaces are approximately regarded as a layer of homogeneous medium with thickness *d*, effective wavenumber *k*, and characteristic acoustic impedance *Z*. Based on the transfer matrix method, the transmission relationships through the hybrid metasurfaces can be derived as

(1) $$\left[\begin{array}{c} p_{t}\\ 0 \end{array}\right]=\Phi T\left[\begin{array}{c} p_{i}\\ p_{r} \end{array}\right]=\frac{1}{4}\left[\begin{array}{cc} T_{11} & {\;T}_{12}\\ {\;T}_{21} & {\;T}_{22} \end{array}\right]\left[\begin{array}{cc} \Phi_{11} & 0\\ 0 & \Phi_{22} \end{array}\right]\left[\begin{array}{c} p_{i}\\ p_{r} \end{array}\right]$$

where *p*_*i*_ and *p*_*r*_ are the complex amplitudes of the incident and reflected sound waves in the water; *p*_*t*_ is the complex amplitude of sound wave transmitted to the air. The transfer matrices **T** and ***Φ*** represent the enhanced transmission by ${\mathrm{MS}}_{1}$ and the phase modulation by ${\mathrm{MS}}_{2}$ with a unitary transmission, respectively, and

(2a) $$\begin{array}{ccc} T_{11} & = & \frac{\left(Z_{m_{1}}+Z_{a}\right)\left(Z_{w}+Z_{m_{1}}\right)}{Z_{m_{1}}Z_{w}}e^{-ik_{m_{1}}d_{m_{1}}}\\ & & +\;\frac{\left(Z_{m_{1}}-Z_{a}\right)\left(Z_{w}-Z_{m_{1}}\right)}{Z_{m_{1}}Z_{w}}e^{ik_{m_{1}}d_{m_{1}}} \end{array}$$

(2b) $$\begin{array}{ccc} T_{12} & = & \frac{\left(Z_{m_{1}}+Z_{a}\right)\left(Z_{w}-Z_{m_{1}}\right)}{Z_{m_{1}}Z_{w}}e^{-ik_{m_{1}}d_{m_{1}}}\\ & & +\;\frac{\left(Z_{m_{1}}-Z_{a}\right)\left(Z_{w}+Z_{m_{1}}\right)}{Z_{m_{1}}Z_{w}}e^{ik_{m_{1}}d_{m_{1}}} \end{array}$$

(2c) $$\begin{array}{ccc} T_{21} & = & \frac{\left(Z_{m_{1}}-Z_{a}\right)\left(Z_{w}+Z_{m_{1}}\right)}{Z_{m_{1}}Z_{w}}e^{-ik_{m_{1}}d_{m_{1}}}\\ & & +\;\frac{\left(Z_{m_{1}}+Z_{a}\right)\left(Z_{w}-Z_{m_{1}}\right)}{Z_{m_{1}}Z_{w}}e^{ik_{m_{1}}d_{m_{1}}} \end{array}$$

(2d) $$\begin{array}{ccc} T_{22} & = & \frac{\left(Z_{m_{1}}-Z_{a}\right)\left(Z_{w}-Z_{m_{1}}\right)}{Z_{m_{1}}Z_{w}}e^{-ik_{m_{1}}d_{m_{1}}}\\ & & +\;\frac{\left(Z_{m_{1}}+Z_{a}\right)\left(Z_{w}+Z_{m_{1}}\right)}{Z_{m_{1}}Z_{w}}e^{ik_{m_{1}}d_{m_{1}}} \end{array}$$

(2e) $$\Phi_{11}=e^{-i\left(k_{a}d_{m_{12}}+k_{m_{2}}d_{m_{2}}\right)},\;\Phi_{22}=e^{i\left(k_{a}d_{m_{12}}+k_{m_{2}}d_{m_{2}}\right)}$$

where the subscripts *w* and *a* indicate the media of water and air, respectively; the subscripts *m*_1_ and *m*_2_ represent ${\mathrm{MS}}_{1}$ and ${\mathrm{MS}}_{2}$, respectively; $d_{m_{1}}$ and $d_{m_{2}}$ are the thickness of ${\mathrm{MS}}_{1}$ and ${\mathrm{MS}}_{2}$, respectively; $d_{m_{12}}$ depicts the distance between ${\mathrm{MS}}_{1}$ and ${\mathrm{MS}}_{2}$; and $Z_{m_{2}}=Z_{a}$ is assumed for simplicity. The detailed derivations of **T** and ***Φ*** can be seen in Note S1, Supporting Information. Then the reflection and transmission coefficients (*R* and *T*) of the hybrid metasurfaces can be easily derived as

(3) $$R=\frac{p_{r}}{p_{i}}=-\frac{T_{21}}{{\;T}_{22}},\;T=\frac{p_{t}}{p_{i}}=\Phi_{11}\frac{T_{11}{\;T}_{22}-T_{12}{\;T}_{21}}{4{\;T}_{22}}$$

The reflection and transmission sound intensity coefficients (*I*_R_ and *I*_T_) can be further calculated by

(4) $$I_{R}=\frac{|p_{r}{|}^{2}}{|p_{i}{|}^{2}}={\left|R\right|}^{2},\;I_{T}=\frac{|p_{t}{|}^{2}}{|p_{i}{|}^{2}}\frac{Z_{w}}{Z_{a}}={\left|T\right|}^{2}\frac{Z_{w}}{Z_{a}}$$

To achieve the full transmission, the reflected sound intensity of the hybrid metasurfaces in the lossless case should be zero (*I*_R_ = 0), that is,

(5) $$\begin{array}{ccc} {\left|T_{21}\right|}^{2} & = & 4\cos^{2}\left(k_{m_{1}}d_{m_{1}}\right)\frac{{\left(Z_{w}-Z_{a}\right)}^{2}}{Z_{w}^{2}}\\ & & +\;4\sin^{2}\left(k_{m_{1}}d_{m_{1}}\right)\frac{{\left(Z_{w}Z_{a}-Z_{m_{1}}^{2}\right)}^{2}}{Z_{w}^{2}Z_{m_{1}}^{2}}=0 \end{array}$$

Since *Z*_*w*_ ≠ *Z*_*a*_, $\cos^{2}\left(k_{m_{1}}d_{m_{1}}\right)$ and ${\left(Z_{w}Z_{a}-Z_{m_{1}}^{2}\right)}^{2}$ should be zero. Therefore, the full‐transmission conditions for the hybrid metasurfaces can be described as

(6) $$k_{m_{1}}d_{m_{1}}=\frac{(2n-1)\pi }{2}\left(n\in Z\right),\;Z_{m_{1}}=\sqrt{Z_{w}Z_{a}},\;Z_{m_{2}}=Z_{a}$$

In this case, the transmission coefficient *T* can be simplified as

(7) $$T=\sqrt{\frac{Z_{a}}{Z_{w}}}e^{-i\left(k_{a}d_{m_{12}}+k_{m_{2}}d_{m_{2}}+k_{m_{1}}d_{m_{1}}\right)}$$

Equation (7) indicates *T* is no longer purely imaginary. The transmitted phase shift under the full transmission can be flexibly adjusted by changing the propagation wavenumber $k_{m_{2}}$ of ${\mathrm{MS}}_{2}$, thus eliminating the limitation of locked phase shift. It should be noted that other solutions can also be found when the constraint of $Z_{m_{2}}=Z_{a}$ is not satisfied, but the strong coupling between ${\mathrm{MS}}_{1}$ and ${\mathrm{MS}}_{2}$ should be considered.^[^ ^3^ ^]^ However, for the case described by Equation (6), ${\mathrm{MS}}_{1}$ and ${\mathrm{MS}}_{2}$ are independent of each other since both can achieve full transmission between different media (water/air and air/air, respectively). This means that the full transmission across dissimilar media can be achieved just by ${\mathrm{MS}}_{1}$, and arbitrary phase manipulation under the full transmission can be further achieved by cascading ${\mathrm{MS}}_{2}$. In this way, the transmission enhancement and phase modulation are decoupled with each other, making it more convenient for the inverse design of hybrid metasurfaces. This also implies that the proposed concept of hybrid metasurfaces for manipulating sound across the water–air interface is feasible in principle.

### 2.2 Decoupled Inverse Design of Hybrid Metasurfaces

To break the confinement of perfect transmission and phase shift, it is only necessary to separately design ${\mathrm{MS}}_{1}$ and ${\mathrm{MS}}_{2}$ based on the above theoretical analysis. Here, we propose a single‐phase solid unit cell to construct ${\mathrm{MS}}_{1}$. The required acoustic impedance is expected to be dynamically satisfied by fully taking advantage of the fluid–solid interactions. Different from previous works,^[^ ^21^, ^22^, ^42^ ^]^ we can avoid strong resonances of tiny air bubbles or narrow air cavities, which may introduce thermoviscous losses to seriously weaken the transmitted sound intensity.^[^ ^21^, ^22^ ^]^ Topology optimization method based on the genetic algorithm^[^ ^50^, ^51^ ^]^ is adopted to find appropriate topological configurations for ${\mathrm{MS}}_{1}$.

The optimization problem for the inverse design of ${\mathrm{MS}}_{1}$ can be described as

(8) $$\begin{array}{ccc} & & \min_{\Omega_{D}}F\left(I_{R},I_{T}^{\prime }\right)=S_{R}I_{R}\left(\Omega_{D}\right)-S_{T}I_{T}^{\prime }\left(\Omega_{D}\right),\\ & & \text{s.t.}\;\Omega_{D}\left(n_{y},n_{z}\right)=1\;\text{or}\;0\;\left(n_{y}\in \left[1,N_{y}\right],n_{z}\in \left[1,N_{z}\right]\right) \end{array}$$

where F denotes the fitness of objective function for the topological distribution Ω_D_; *I*_R_(Ω_D_) is the reflection sound intensity coefficient without viscosity losses; $I_{T}^{\prime }\left(\Omega_{D}\right)$ is transmitted sound intensity coefficient with viscosity losses; *S*_R_ = 200 and *S*_T_ = 100 represent the weight coefficients of *I*_R_(Ω_D_) and $I_{T}^{\prime }\left(\Omega_{D}\right)$, respectively; Ω_D_(*n*_*y*_, *n*_*z*_) = 1 or 0 indicates the solid (1) or air (0), respectively; and *N*_*y*_ = 24 and *N*_*z*_ = 12 are the numbers of discrete pixels in the width and thickness directions, respectively. It is noted that *I*_R_(Ω_D_) and $I_{T}^{\prime }\left(\Omega_{D}\right)$ instead of $Z_{m_{1}}$ and $k_{m_{1}}$ is involved in the optimization just for convenience. The retrieving method for *I*_R_(Ω_D_) and $I_{T}^{\prime }\left(\Omega_{D}\right)$ is presented in Note S2, Supporting Information. Meanwhile, the smoothing process by chamfering is employed inside ${\mathrm{MS}}_{1}$ unit cells to improve the convergence of finite element calculation. The target frequency is selected as 10 kHz, and the width and thickness of ${\mathrm{MS}}_{1}$ unit are *a* = 2/3λ_*a*_ and $d_{m_{1}}=\lambda_{a}/3$ , respectively, with λ_*a*_ being the wavelength in air. It is noted that it is very difficult to achieve full transmission via ${\mathrm{MS}}_{1}$ with loss. By finding the optimal solution to Equation (8), the optimized ${\mathrm{MS}}_{1}$ with or without loss promises the highest possible sound transmission at the water–air interface. This will facilitate proof of principle and experimental observations.

Regarding the inverse design of ${\mathrm{MS}}_{2}$, two‐bit coding unit cells with unitary transmission are expected. 3/10π, 8/10π, 13/10π, and 18/10π phase shifts (a phase interval of π/2, encoded as 00, 01, 10, and 11) are chosen for ease of optimization. The optimization problem for ${\mathrm{MS}}_{2}$ can be described as

(9) $$\begin{array}{ccc} & & \min_{\Omega_{D}}F\left(\phi ,t\right)=\left\{\begin{array}{c} S_{\phi }{\left|\phi -\phi_{c}\right|}^{2}/{\left(2\pi \right)}^{2}+S_{p}\left(t\right),\left|t\right|<0.5\\ S_{\phi }{\left|\phi -\phi_{c}\right|}^{2}/{\left(2\pi \right)}^{2}-S_{t}{\left|t\right|}^{2},\;\left|t\right|\geq 0.5 \end{array}\right.\\ & & \text{s.t.}\;\Omega_{D}\left(n_{y},n_{z}\right)=1\;\text{or}\;0\;\left(n_{y}\in \left[1,N_{y}\right],n_{z}\in \left[1,N_{z}\right]\right), \end{array}$$

where ϕ and *t* are the phase and transmission of unit cell, respectively; ϕ_c_ is the target phase shift of each coding unit cell; *S*_ϕ_ = 4000 and *S*_*t*_ = 10 represent the weight coefficients of phase and transmission, respectively; and *S*_p_(*t*) = 500 is a penalty term imposed on the transmission when |*t*| < 0.5 to facilitate simultaneous optimization of ϕ and *t*. The detailed calculation and optimized process for ${\mathrm{MS}}_{1}$ and ${\mathrm{MS}}_{2}$ are illustrated in Note S3, Supporting Information.

**Figure 2:**
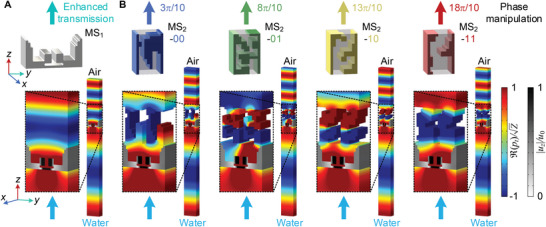
Decoupled inverse design of hybrid metasurfaces based on the topology optimization. Panel (A) shows the geometrical configuration of the optimized ${\mathrm{MS}}_{1}$ unit cell and the corresponding water‐to‐air sound transmission calculation for ${\mathrm{MS}}_{1}$ unit cell under periodic conditions. Panel (B) presents the geometrical configurations of four optimized ${\mathrm{MS}}_{2}$ unit cells (labeled as 00, 01, 10, and 11) and the corresponding water‐to‐air sound transmission calculation for hybrid ${\mathrm{MS}}_{1}$ and ${\mathrm{MS}}_{2}$ unit cells under periodic conditions. The thicknesses of ${\mathrm{MS}}_{1}$ and ${\mathrm{MS}}_{2}$ unit cells are $d_{m_{1}}=\lambda_{a}/3$ and $d_{m_{2}}=\lambda_{a}/2$, respectively. In the calculation, the distance between ${\mathrm{MS}}_{1}$ and ${\mathrm{MS}}_{2}$ unit cells is set as $d_{m_{12}}=\lambda_{a}/100$ and the solid parts of ${\mathrm{MS}}_{2}$ unit cells are treated as hard boundaries due to the huge impedance contrast between the air and solid. The perfect transmission with expected phase shifts (from 3π/10 to 18π/10 with an interval of π/2) is obtained through the enhanced transmission of ${\mathrm{MS}}_{1}$ unit and phase modulation of four coding unit cells. The results show the normalized real part of total sound pressure (*p*_*t*_) by square root of acoustic impedance ($\sqrt{Z}$) of host medium (water or air) and normalized displacement amplitude field |*u*_*z*_|/*u*_0_ of ${\mathrm{MS}}_{1}$ solid structure along the *z*‐axis.

**Figure** **2** gives the optimized geometrical configurations for the ${\mathrm{MS}}_{1}$ and ${\mathrm{MS}}_{2}$ unit cells and the corresponding water‐to‐air sound transmission. The normalized sound pressure field shown in Figure 2A suggests that perfect sound transmission between water and air is achieved by the optimized ${\mathrm{MS}}_{1}$ unit cell. In addition, we further calculate the effective acoustic impedance and wavenumber of ${\mathrm{MS}}_{1}$ unit cell. The strict impedance‐matching conditions given by Equation (6) for ${\mathrm{MS}}_{1}$ can be well satisfied (see Note S4, Supporting Information). It indicates that the quarter‐wave impedance transformer between water and air is successfully designed by the proposed optimization strategy. Figure 2B shows the water‐to‐air sound transmission when the four optimized unit cells of ${\mathrm{MS}}_{2}$ are hybridized with ${\mathrm{MS}}_{1}$ unit cell, respectively, where the distance $d_{m_{12}}$ between them is only λ_*a*_/100. As expected, the hybrid ${\mathrm{MS}}_{1}$ and ${\mathrm{MS}}_{2}$ exhibit nearly full sound transmission from water to air and provide the expected phase shifts at the same time. The perfect transmission with phase shift is mainly attributed to the weak coupling between ${\mathrm{MS}}_{1}$ and ${\mathrm{MS}}_{2}$ unit cells resulting from the air gap (see Note S5, Supporting Information). This guarantees the feasibility of individual design for ${\mathrm{MS}}_{2}$ units and may allow ${\mathrm{MS}}_{2}$ to achieve the nonlocal design with perfect transmission based on the lattice diffraction theory.^[^ ^33^, ^52^ ^]^

### 2.3 Experimental Verification

Due to the decoupled modulation of sound for ${\mathrm{MS}}_{1}$ and ${\mathrm{MS}}_{2}$, the enhanced transmission performance of ${\mathrm{MS}}_{1}$ will be checked independently. The mechanism of enhancing water–air acoustic transmission will be revealed through vibration tests. Wavefront manipulation under enhanced transmission through hybrid metasurfaces will then be demonstrated.

#### 2.3.1 Enhanced Water‐to‐Air Sound Transmission

**Figure 3:**
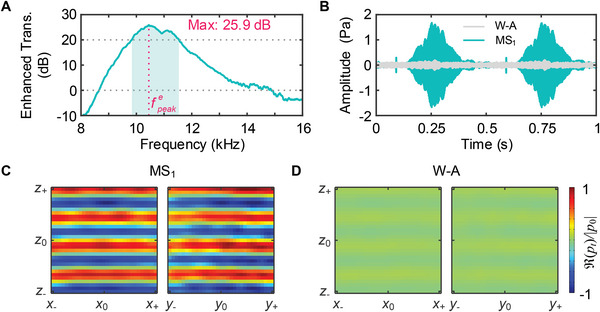
Experimental demonstration of enhanced water‐to‐air sound transmission. Panel (A) presents the measured sound amplitude enhancement (dB) of ${\mathrm{MS}}_{1}$ compared to the bare water–air interface, where the shadow area indicates the frequency span (from 9.84 to 11.53kHz) with over 20 dB enhancement. The maximal transmission enhancement is ≈25.9 dB at the peak frequency $f_{\text{peak}}^{e}=10.45\;\mathrm{kHz}$. Panel (B) shows the measured time‐domain sound signals from water to air through the ${\mathrm{MS}}_{1}$ and water–air interface (W‐A) under a broadband pulse incidence from 8 to 16 kHz. Panels (C) and (D) present the measured real parts of transmitted sound fields in *xz*‐ and *xy*‐planes from water to air through the ${\mathrm{MS}}_{1}$ and water–air interface at 10.45kHz, respectively, where $|p_{0}|=0.5\;\mathrm{Pa}$, $z_{0}\approx \;113.3\;\mathrm{mm}$, $x_{0}=y_{0}=0\;\mathrm{mm}$, $z_{\pm }=z_{0}\pm 60\;\mathrm{mm}$, $x_{\pm }=x_{0}\pm 60\;\mathrm{mm},$ and $y_{\pm }=y_{0}\pm 60\;\mathrm{mm}$.

Figure 1B shows the photography of the experimental setup for water‐to‐air acoustic experiments. The photograph of the fabricated ${\mathrm{MS}}_{1}$ sample with a partially enlarged view of the cross‐section is shown in Figure 1C. Note that ${\mathrm{MS}}_{2}$ (with axial focusing function as an example) shown in Figure 1D is not involved in the enhanced water‐to‐air sound transmission experiment. A broadband pulse with bandwidth from 8 to 16 kHz is emitted by the underwater transducer. The transmission amplitude enhancement (ET) by ${\mathrm{MS}}_{1}$ with respect to the bare water–air interface is calculated by

(10) $$\text{ET}=20\log_{10}\sqrt{\sum_{i=1}^{I}\sum_{j=1}^{J}{\left|p_{ij}^{{\mathrm{MS}}_{1}}\right|}^{2}/\sum_{i=1}^{I}\sum_{j=1}^{J}{\left|p_{ij}^{W-A}\right|}^{2}}\;\;\;\left(\mathrm{dB}\right)$$

where $|p_{ij}^{{\mathrm{MS}}_{1}}|$ and $|p_{ij}^{W-A}|$ are the measured sound pressure amplitudes at each sampling point through ${\mathrm{MS}}_{1}$ and the water–air interface, respectively; and *I* = *J* = 21 represents the numbers of sampling points along the *x* and *y*‐axes in a 20 cm × 20 cm area (*xy*‐plane) above the center of ${\mathrm{MS}}_{1}$. **Figure** **3**A plots the variation of ET as a function of frequency. The measured transmission amplitudes through the ${\mathrm{MS}}_{1}$ and bare water–air interface are presented in Note S6, Supporting Information. It can be observed that ${\mathrm{MS}}_{1}$ exhibits above 20 dB (ten times) amplitude enhancement from 9.84 to 11.53 kHz. The measured peak frequency is found to be around $f_{\text{peak}}^{e}=10.45$ kHz with ≈25.9 dB (nearly 20 times) transmission enhancement, close to the perfect transmission of 30 dB. The time‐domain sound signals received at a test point, as shown in Figure 3B, further exhibit the sharp contrast between sound pressure amplitudes transmitted by ${\mathrm{MS}}_{1}$ and the bare water–air interface. A more intuitive presentation is also provided in Movies S1 and S2, Supporting Information. They dynamically demonstrate the enhancement (reduction) of the sound pressure when ${\mathrm{MS}}_{1}$ is placed on (removed from) the water surface under broadband pulse or peak frequency incidence.

Furthermore, we measure the sound pressure fields transmitted through ${\mathrm{MS}}_{1}$ and bare water–air interface at the peak frequency $f_{\text{peak}}^{e}=10.45\;\mathrm{kHz}$, as shown in Figure 3C,D, respectively. It can be clearly observed that the sound waves emitted from underwater transducer are converted into uniform plane waves after passing through ${\mathrm{MS}}_{1}$, and the transmission amplitudes through ${\mathrm{MS}}_{1}$ over the entire measurement area are greatly improved compared to the bare water–air interface. This mainly benefits from the transmission enhancement of ${\mathrm{MS}}_{1}$ under wide‐angle incidence. According to Snell's law (*k*_w_sin θ_w_ = *k*_a_sin θ_*a*_ with θ_w_ and θ_a_ being incident angle in water and refracted angle in air, respectively), the transmitted angles in air are less than 13.2° within ±90° oblique incidences from water. Quantitative analysis for transmission enhancement of ${\mathrm{MS}}_{1}$ under oblique incidences is presented in Note S7, Supporting Information. It suggests that ${\mathrm{MS}}_{1}$ can maintain over 98.05% sound transmission within ±60° oblique incidence. These enhanced plane waves in air are very helpful for the subsequent phase manipulation by the hybrid ${\mathrm{MS}}_{2}$. In addition, we also measure the sound fields near the peak frequency (11 kHz). Similar enhanced performance is also clearly observed, see Note S8, Supporting Information. The measured peak frequency slightly deviates from the simulated one $f_{\text{peak}}^{s}=10\;\mathrm{kHz}$. This may originate from deviations in material parameters. As we have checked, the peak frequency will shift upward as Young's modulus of ${\mathrm{MS}}_{1}$ increases, see Note S9, Supporting Information. Overall, the experimental results effectively validate the excellent performance of ${\mathrm{MS}}_{1}$ for enhanced sound transmission across the water–air interface.

#### 2.3.2 Mechanism Demonstration of Enhanced Sound Transmission

**Figure 4:**
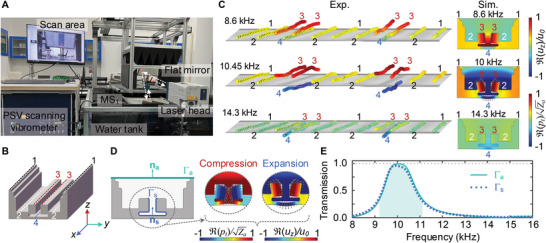
Mechanism demonstration for sound transmission enhancement across the water–air interface. Panel (A) shows the photograph of the experimental set‐up for vibration measurements of ${\mathrm{MS}}_{1}$. Panel (B) presents the four different measured locations on a unit cell, labeled as positions 1, 2, 3, and 4, respectively. Panel (C) presents the measured vibration at locations 1–4 on ${\mathrm{MS}}_{1}$ unit cells at 8.6 kHz ($f<f_{\text{peak}}^{e}$), 10.45 kHz ($f=f_{\text{peak}}^{e}$), and 14.3 kHz ($f>f_{\text{peak}}^{e}$), where the color map plots the normalized displacement *u*_*z*_/|*u*_0_| along the *z*‐axis direction. The corresponding simulated results at 8.6 kHz ($f<f_{\text{peak}}^{s}$), 10 kHz ($f=f_{\text{peak}}^{s}$), and 14.3 kHz ($f>f_{\text{peak}}^{s}$) are shown on the right for comparison. The boundaries for the energy flux calculation are marked in panel (D), where the inner solid boundary at the bottom of ${\mathrm{MS}}_{1}$ unit cell and outer air boundary at the surface are labeled as Γ_s_ and Γ_a_, respectively, and the arrows **n**_s_ and **n**_a_ indicate the corresponding normal vectors at the boundaries. The insets on the right show the compression and expansion of the inner air cavity induced by the flexural deformations of solid boundaries. Panel (E) shows the water‐to‐air sound pressure transmission as a function of frequency calculated by integrating the energy flux at the solid and air boundaries Γ_s_ and Γ_a_.

**Figure** **4**A shows the photograph of the experimental setup for the vibration test of ${\mathrm{MS}}_{1}$. Four different positions (labeled as positions 1, 2, 3, and 4, respectively) on one unit cell are chosen as the test points, as shown in Figure 4B. Figure 4C presents the measured vibrations of ${\mathrm{MS}}_{1}$ at different frequencies *f*, and the corresponding simulated results of a single unit cell are given on the right for comparison. A complete animation is provided in Movie S4, Supporting Information. Good agreement is observed between the measured and simulated results. Vibrations at the notch (positions 3 and 4) are more pronounced and significantly stronger than on both sides (positions 1 and 2) when $f=f_{\text{peak}}^{e}$ (or $f_{\text{peak}}^{s}$). At the same time, an anti‐phase vibration mode can be clearly observed at the masses (position 3) and the thin wall (position 4), which will induce a resonance state of compression and expansion in the inner air cavity. In this case, the collaborative resonance of the masses and thin wall at positions 3 and 4 seems to act as an energy converter, absorbing the underwater acoustic energy and then radiating it into the air. However, when the incident frequency is away from the peak one, such as 8.6 and 14.3 kHz, the collaborative vibration at the notch becomes unapparent. So, the enhanced water‐to‐air sound transmission by ${\mathrm{MS}}_{1}$ may be attributed to the unique flexural vibrations of the solid unit cell, which gives rise to the required sound impedance by inducing compression and expansion of the inner air cavity.

Quantitative analysis is further made by comparing the outward mechanical energy flux generated by the solid vibration at the notch and the total outward sound energy flux on the unit cell surface. The boundaries (Γ_*s*_ and Γ_*a*_) for calculating the energy flux and the corresponding unit normal vectors are marked in Figure 4D. The outward energy flux *E*_s_ induced by the flexural vibration and water‐to‐air sound energy flux *E*_a_ are calculated by

(11) $$E_{s}=\int_{\Gamma_{s}}\left[S_{x},S_{y}\right]\cdot n_{s}\mathrm{ds},\;E_{a}=\int_{\Gamma_{a}}\left[I_{x},I_{y}\right]\cdot n_{a}\mathrm{ds},$$

where Sm=12Re(−σmnutn∗) and Im=12Re(pmvm∗) with *m* = *n* = *x*, *y* representing the components of mechanical energy flux and sound intensity along *x* or *y* axis, respectively; σ and *u*_t_ are the stress tensor and velocity field of the solid domain, respectively; *p* and *v* are the sound pressure and velocity of air domain, respectively; and Re and * represent the real part and conjugate operations, respectively. Figure 4E plots the water‐to‐air transmission $T_{s}=\sqrt{E_{s}/E_{0}}$ and $T_{a}=\sqrt{E_{a}/E_{0}}$ at the boundaries Γ_s_ and Γ_a_, where $E_{0}=a{\left|p_{i}\right|}^{2}/\left(2Z_{w}\right)=7.62\times 10^{-9}\;W\;m^{-1}$ $\left(|p_{i}|=1\;\mathrm{Pa}\right)$ represents the incident energy. It can be seen that the maximal outward mechanical energy flux from solid vibrations is also concentrated near the peak frequency, showing a good consistency with the sound energy flux curve. This suggests that almost all acoustic energy transmission from water to air is contributed by the solid vibrations at the notch. It is noted that there is a small amplitude deviation between the both because the solid vibrations far from the notch are not taken into account.

#### 2.3.3 Enhanced Water–Air Sound Wavefront Manipulation

**Figure 5:**
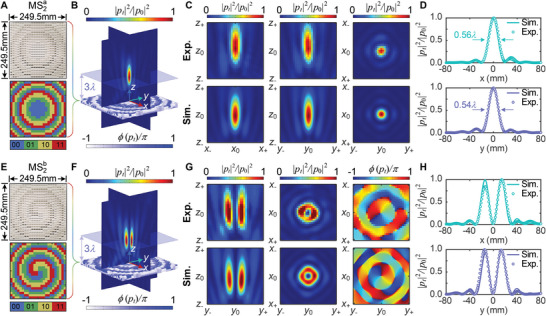
Experimental demonstration of enhanced sound axial focusing and vortex beams focusing across the water–air interface by hybrid metasurfaces. Panels (A) and (E) show the photographs of fabricated samples of ${\mathrm{MS}}_{2}^{a}$ and ${\mathrm{MS}}_{2}^{b}$ for axial focusing and vortex beam self‐focusing with the corresponding binary coding phase distributions, respectively. Enlarged front and back views of ${\mathrm{MS}}_{2}^{a}$ and ${\mathrm{MS}}_{2}^{b}$ samples are clearly presented in Figure 1D and Figure S14, Supporting Information, respectively. Both samples consist of 24 × 24 unit cells. The numbers 00, 01, 10, and 11 represent the unit cells with phase shifts of 3/10π, 8/10π, 13/10π, and 18/10π, respectively. Panels (B) and (F) show the 3D full‐wave simulated intensity fields of ${\mathrm{MS}}_{2}^{a}$ and ${\mathrm{MS}}_{2}^{b}$ from air to air for sound axial focusing and vortex beam self‐focusing, respectively, where the normalized pressure |*p*_0_| is chosen as 11|*p*_*i*_| and 6.2|*p*_*i*_| in panels (B) and (F) for simulation, respectively. Panels (C) and (G) present the measured sound fields from water to air in different planes through hybrid ${\mathrm{MS}}_{1}$ and ${\mathrm{MS}}_{2}$ at 11kHz for axial focusing and vortex beams focusing, respectively. The corresponding simulated results obtained from panels (B) and (F) are also given for comparison. The normalized pressure |*p*_0_| is chosen as 4.1and 2.5Pa in panels (C) and (G) for experiments, respectively. $z_{0}=3\lambda_{a}=93.5\;\mathrm{mm}$, $x_{0}=y_{0}=0\;\mathrm{mm}$, $z_{\pm }=z_{0}\pm 60\;\mathrm{mm}$, $x_{\pm }=x_{0}\pm 60\;\mathrm{mm},$ and $y_{\pm }=y_{0}\pm 60\;\mathrm{mm}$. Panels (D) and (H) plot the measured and simulated intensity distributions on two transverse lines along *x*‐axis and *y*‐axis in *xy*‐plane through the point (0, 0, 3λ_a_) for axial focusing and vortex beams focusing, respectively.

We start with the experimental demonstration of enhanced 3D axial sound focusing by hybrid metasurfaces. For convenience, the operating frequency of hybrid metasurfaces is chosen at $f_{m}=11\;\mathrm{kHz}$ near the peak frequency. The ideal phase distribution Ψ_F_ on the exit surface of ${\mathrm{MS}}_{2}$ can be expressed as ^[^ ^53^, ^54^ ^]^

(12) $$\begin{array}{c} \Psi_{F}\left(x,y\right)=k_{a}\left[\sqrt{{\left(x-x_{0}\right)}^{2}+{\left(y-y_{0}\right)}^{2}+F_{0}^{2}}-\sqrt{x_{0}^{2}+y_{0}^{2}+F_{0}^{2}}\right] \end{array}$$

where (*x*_0_, *y*_0_) = (0, 0) is the center of ${\mathrm{MS}}_{2}$; and the focal length *F*_0_ is chosen as $3\lambda_{a}\approx 93.5\;\mathrm{mm}$ with λ_a_ being the wavelength in air. Following the principle of proximity, the ideal phase distribution Ψ_F_ is approximated by the two‐bit coding unit cells with unitary transmission and phase shifts of 3π/10, 8π/10, 13π/10, and 18π/10, respectively. **Figure** **5**A depicts the photograph of fabricated sample ${\mathrm{MS}}_{2}^{a}$ for axial focusing with the corresponding coding phase sequence. It can be seen that the encoded phase sequence is centrosymmetric and has a parabolic gradient distribution along the radial direction, which can ensure that the transmitted sound waves are concentrated in the axial direction of ${\mathrm{MS}}_{2}$.

To better demonstrate the focusing characteristics, we perform numerical simulations on the air‐to‐air transmitted sound field through ${\mathrm{MS}}_{2}^{a}$. The background field with uniform plane wavefronts is used as the incidence. Figure 5B presents the simulated normalized sound intensity distributions on the *xz*‐ and *yz*‐planes. Excellent focusing performance is exhibited in the prescribed focal point, showing the good ability of ${\mathrm{MS}}_{2}^{a}$ on phase modulation. The measured normalized intensity fields and the corresponding simulated results (obtained from Figure 5B) on the three orthogonal planes are illustrated in Figure 5C, and the measured real parts are presented in Note S10, Supporting Information. As expected, the sound waves emitted from underwater transducer are well gathered around the prescribed focus after transmission enhancement and phase manipulation of the hybrid ${\mathrm{MS}}_{1}$ and ${\mathrm{MS}}_{2}^{a}$. The measured sound amplitude at the preset spot (*z* = 3λ_a_) is enhanced by nearly 42 dB (about 125 times) compared with the bare water–air interface, showing the distinguished focusing features (see Movie S3, Supporting Information). To further quantitatively characterize the focusing performance, we measure the pressure intensity distributions along *x*‐ and *y*‐axis directions through the prescribed focal spot, and plot the normalized results in Figure 5D. It can be seen as a good agreement with the simulated one. The full width at half‐maximum (FWHM) in the *x*‐ and *y*‐axis directions are about 0.56λ_a_ and 0.54λ_a_, respectively, close to the diffraction limit of 0.5λ_a_. In addition, we also measure the pressure intensity distribution on the central axis along *z*‐axis. It is found that the actual focal length is slightly larger than the preset one. This may be due to the fact that unavoidable boundary reflections inside water tank result in inhomogeneous amplitudes of transmitted sound field from water to air. Overall, the experimental and simulated results verify the enhanced axial focusing performance of hybrid metasurfaces across the water–air interface.

It is emphasized that the principle of phase modulation under enhanced transmission is general and applicable to other complex wavefront manipulation across the water–air interface, such as vortex fields with orbital angular momentum (OAM). OAM is regarded as an additional spatial degree of freedom to promote the channel capacity of acoustic communication owing to the perfect orthogonality between different topological charges.^[^ ^55^ ^]^ Benefiting from the decoupled modulation by hybrid metasurfaces, the realization of desired sound fields with the enhanced transmission is just required to replace the phase‐encoded sequence of ${\mathrm{MS}}_{2}$ rather than redesign new unit cells. To generate the vortex beams with spiral wavefronts, the required phase distribution Ψ_V_ on ${\mathrm{MS}}_{2}$ can be expressed as^[^ ^56^ ^]^

(13) $$\Psi_{V}\left(x,y\right)=L_{V}\cdot \arctan\left(y/x\right)$$

where *L*_V_ represents the expected topological charge of OAM. However, the vortex beams propagating in free space are inherently divergent. To extend the sound energy to the desired location, we consider the generation of self‐focusing vortex beams. The total phase distribution Ψ_*VF*_ can be obtained by superposing a parabolic phase profile along the radial direction according to the digital convolution theorem,^[^ ^56^, ^57^ ^]^ namely

(14) $$\Psi_{\text{VF}}\left(x,y\right)=\Psi_{V}\left(x,y\right)+\Psi_{F}\left(x,y\right)$$

Here, OAM with *L*_V_ = 1 and focus length *F*_0_ = 3λ_a_ are chosen for demonstration.

Figure 5E shows the fabricated sample of ${\mathrm{MS}}_{2}^{b}$ for vortex beam self‐focusing with the corresponding encoding‐phase sequence. It can be observed that the spiral phase variation around the center superposes a non‐uniform gradient distribution along the radial direction to effectively gather the generated sound vortex beam. The simulated intensity fields on *xz*‐ and *yz*‐planes from air to air by ${\mathrm{MS}}_{2}^{b}$ are depicted in Figure 5F. Different from one main lobe of the axial focusing shown in Figure 5B, the sound energy of the self‐focusing vortex is split into two lobes at each cross‐section. This is mainly because of the singularity of the helical phase at the center, which results in a null sound intensity at the center axis. Figure 5G shows the measured and simulated intensity fields on *yz*‐ and *xy*‐planes and phase distribution on *xy*‐plane. The measured real parts of transmitted sound fields are presented in Note S10, Supporting Information. Two high‐intensity beams can be clearly observed apart from the central axis on *xy*‐plane. Meanwhile, the intrinsic features of vortex beams, such as ring‐shaped intensity field and spiral phase distribution, are also clearly observed from the measured and simulated results on *xy*‐plane (*z* = 3λ_a_). Furthermore, we also measure the sound intensity distributions along *x*‐ and *y*‐axis directions at *z* = 3λ_a_ and plot the results in Figure 5H. Different from axial focusing, the amplitude valleys are located at the center and the sound intensity is concentrated on the two peaks. This shows good consistency with the simulated results. In addition, slight deviations between measured and simulated phase distributions in Figure 5G can be also noticed, possibly due to the differences in incident waves.

Furthermore, we consider the generation of OAM with anisometric radius and higher topological charge from water to air, which is beneficial to acoustic communication with high channel capacity. In this case, we take advantage of the energy concentration of the non‐diffraction Bessel beam to reduce the diffusion of the vortex beam in free space. By superposing a uniform gradient phase Ψ_B_ for generating Bessel beam, the final phase distribution Ψ_VB_ implemented on ${\mathrm{MS}}_{2}$ can be expressed as^[^ ^56^ ^]^

(15) $$\Psi_{\mathrm{VB}}\left(x,y\right)=\Psi_{V}\left(x,y\right)+\Psi_{B}\left(x,y\right)$$

where $\Psi_{B}\left(x,y\right)=k_{a}\sqrt{{\left(x-x_{0}\right)}^{2}+{\left(y-y_{0}\right)}^{2}}\sin\theta_{B}$ with θ_B_ being the cone angle of Bessel beam. In addition, for the generation of a vortex beam with anisometric OAM, the phase profile Ψ_V_ can be extended as

(16) $$\Psi_{V}^{\prime }\left(x,y\right)=L_{V}\cdot \arctan\left(\xi y/x\right)$$

where ξ represents the anisometric factor of OAM. By setting ξ other than one, the spiral phase distribution of vortex beam at the center can be switched from a circle to an ellipse, which may bring a new degree of freedom to manipulate the vortex beams.

**Figure 6:**
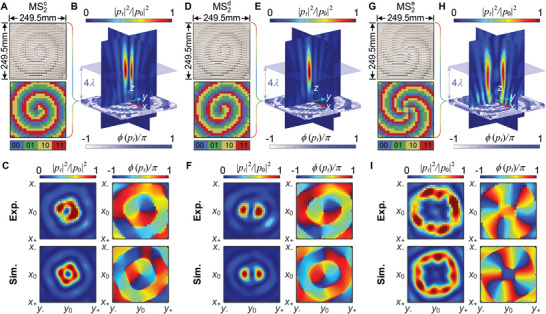
Experimental demonstration of generating various non‐diffracting sound vortex beams across the water–air interface by hybrid metasurfaces. Panels (A), (D), and (G) show the photographs of fabricated samples of ${\mathrm{MS}}_{2}^{c}$, ${\mathrm{MS}}_{2}^{d}$, and ${\mathrm{MS}}_{2}^{e}$ with the corresponding binary coding phase distributions for generating non‐diffracting vortex beams with the first‐order circular OAM, the first‐order ellipse OAM and the fourth‐order circular OAM, respectively, which both consist of 24 × 24 unit cells. Enlarged front and back views of ${\mathrm{MS}}_{2}^{c}$, ${\mathrm{MS}}_{2}^{d}$, and ${\mathrm{MS}}_{2}^{e}$ samples are clearly presented in Figure S14, Supporting Information. Panels (B), (E), and (H) show the corresponding 3D full‐wave simulated intensity fields from air to air of ${\mathrm{MS}}_{2}^{c}$, ${\mathrm{MS}}_{2}^{d}$, and ${\mathrm{MS}}_{2}^{e}$ for generating the three types of non‐diffracting sound vortex beams, respectively, where the normalized pressure |*p*_0_| is chosen as 4.5|*p*_*i*_|, 5.6|*p*_*i*_|, and 2.8|*p*_*i*_| in panels (B), (E), and (H) for simulation, respectively. Panels (C), (F), and (I) present the measured sound intensity and phase fields from water to air in *xy*‐plane (*z* = 4λ_a_) through hybrid ${\mathrm{MS}}_{1}$ and ${\mathrm{MS}}_{2}$ at 11kHz for the three types of vortex beams, respectively. The normalized pressure |*p*_0_| is chosen as 1.8, 2.2, and 1.08Pa in panels (C), (F), and (I) for experiments, respectively. $z_{0}=4\lambda_{a}=124.7\;\mathrm{mm}$, $x_{0}=y_{0}=0\;\mathrm{mm}$, $z_{\pm }=z_{0}\pm 60\;\mathrm{mm}$, $x_{\pm }=x_{0}\pm 60\;\mathrm{mm},$ and $y_{\pm }=y_{0}\pm 60\;\mathrm{mm}$.

For demonstration, we construct ${\mathrm{MS}}_{2}^{d}$ to generate a non‐diffraction vortex beam carrying the first‐order anisometric OAM. Meanwhile, ${\mathrm{MS}}_{2}^{c}$ used to generate the standard first‐order OAM is also constructed for comparison. The anisometric factor and the cone angle of the Bessel beam are chosen as ξ = 3 and $\sin\theta_{B}=2/\sqrt{13}$. The fabricated samples of ${\mathrm{MS}}_{2}^{c}$ and ${\mathrm{MS}}_{2}^{d}$ with the corresponding encoded phase sequences for the standard and anisometric are shown in **Figure** **6**A,D, respectively. Compared with the standard spiral phase in Figure 6A, the spiral phase distribution in Figure 6D is apparently stretched to an elliptic shape to carry non‐standard OAM. Besides, both phase distributions contain a uniform gradient variation along a radial direction to generate the non‐diffraction beam. Figure 6B,E presents the simulated sound intensity fields from air to air of ${\mathrm{MS}}_{2}^{c}$ and ${\mathrm{MS}}_{2}^{d}$, respectively. It can be clearly observed from Figure 6B that a hollow beam with less divergent propagation is formed around 4λ_a_. The sound energy is gathered along the central axis and almost evenly distributed on *xz*‐ and *yz*‐planes. In contrast, the energy for the anisometric OAM in Figure 6E is only concentrated on the *yz*‐plane due to ξ > 1. The measured and simulated sound intensity and phase distributions for the two cases on *xy*‐plane at *z* = 4λ_a_ are shown in Figure 6C,F, respectively, and the corresponding real parts of sound fields are presented in Note S10, Supporting Information. As expected, we can clearly observe that the sound energy for the standard OAM is concentrated on a ring, while the anisometric OAM is concentrated in one direction along the *x*‐axis. The vortex phase in Figure 6F also exhibits an elliptic pattern different from the circular one shown in Figure 6C. The intriguing features are mainly owing to the introduction of an anisometric factor, which changes the sweeping velocity of the phase along the circumferential direction.

Next, we design hybrid metasurfaces to generate a standard fourth‐order OAM (ξ = 1 and $\sin\theta_{B}=2/\sqrt{13}$) across the water–air interface. Figure 6G presents the fabricated sample of ${\mathrm{MS}}_{2}^{e}$ with the corresponding encoded phase sequence. The simulated intensity field from air to air is presented in Figure 6H, exhibiting the hollow beam with non‐diffractive propagation. Then the measured and simulated intensity and phase fields on *xy*‐plane at *z* = 4λ_*a*_ are shown in Figure 6I. Four spiral phase patterns and the ring‐shaped energy distribution are also clearly observed, thus validating the proposed concept for cross‐media transmission manipulation.

## 3 Conclusions

In this work, we theoretically propose and experimentally validate the concept of hybrid metasurfaces for the transmission enhancement and phase manipulation of sound across the water–air interface. An inverse‐design strategy based on the topology optimization is systematically developed to separately design the hybrid metasurfaces. To achieve perfect transmission between water and air, ${\mathrm{MS}}_{1}$ is successfully optimized to have an effective acoustic impedance of about 60 times that of air. Experimental results suggest that ≈25.9 dB (nearly 20 times) transmission enhancement across the water–air interface is obtained by ${\mathrm{MS}}_{1}$ at the peak frequency. The physical mechanism of enhanced transmission is that the fluid–solid interactions induce the specific vibration modes of ${\mathrm{MS}}_{1}$ to dynamically satisfy the required impedance‐matching condition. The optimized ${\mathrm{MS}}_{1}$ also exhibits good performance of broadband and wide‐angle transmission enhancement. In addition, the water‐to‐air perfect transmission is further achieved by a thinner ${\mathrm{MS}}_{1}$ (whose thickness is 1/10 of the wavelength in the air). When the losses are considered, more than 50% energy transmission can also be obtained (see Note S11, Supporting Information).

Furthermore, four discrete unit cells with unitary transmission and π/2 phase shift interval are inversely optimized to construct the coding ${\mathrm{MS}}_{2}$ with wavefront manipulation ability. Based on the digital convolution theorem, various customized sound fields are well realized by modifying the encoded phase sequences on ${\mathrm{MS}}_{2}$. Cross‐media (water‐to‐air) experiments also fully demonstrate that the sound waves in the water are effectively transmitted to the air and reshaped as the desired distributions through the hybrid metasurfaces. Nearly 42 dB (about 125 times) amplitude enhancement is experimentally observed at the preset focus through the hybrid metasurfaces with axial focusing function. Meanwhile, the ability to generate arbitrary sound vortex beams from water to air is also explored. Good agreements between the experimental and simulated results effectively validate the flexibility and universality of hybrid modulations.

Generally, the presented inverse‐design strategy allows us to customize the structure design on demand, such as different operating frequencies, thinner thickness, etc. Meanwhile, the separate implementation of hybrid metasurfaces will also bring more flexibility and expansibility. Controllable digital‐coding metasurfaces^[^ ^57^, ^58^ ^]^ can be allowed to be integrated with the ${\mathrm{MS}}_{1}$. This will play a significant role in some potential and promising engineering applications such as high‐speed ocean‐air wireless communications, etc. In addition, the proposed strategy and model for cross‐media wave manipulation can also be applied to other dissimilar media, such as air/solid, water/solid, or different solids, thus beneficial to the design of novel trans‐media wave devices.

## 4 Experimental Section

### Numerical Simulations

The numerical simulations were performed with the commercial finite element software, COMSOL Multiphysics. For the water‐to‐air sound transmission calculation in Figure 2, pressure acoustics and solid mechanics modules were applied to solve the sound pressure and displacement fields, respectively. The interfaces between water/air and solid were imposed on acoustic‐structure boundary conditions, that is, the continuity of the normal acceleration and traction with the vanishing of tangential traction at the interfaces. Floquet periodic boundary conditions were applied to the lateral boundaries of the calculation domains. Perfectly matched layers were employed to the top and bottom to eliminate boundary reflections. A uniform plane wave background field was used as the incident wave and applied to the water domain at the bottom of ${\mathrm{MS}}_{1}$. 3D quadratic Lagrange (serendipity) elements for acoustics (solids) were built by sweeping the free triangular meshes on the cross section. The maximum element sizes of the ${\mathrm{MS}}_{1}$ and ${\mathrm{MS}}_{2}$ unit cells, air, and water domains were taken as λ_a_/144 (≈1/4 the height of the pixel grid), λ_a_/10, and λ_w_/40, respectively, to ensure the accuracy of the numerical calculations. For the simulated vibration modes shown in Figure 4C, the results were obtained by extracting the displacement fields in the calculation of the sound intensity coefficient for ${\mathrm{MS}}_{1}$ at different frequencies (see Note S2, Supporting Information). For the full‐wave simulations on ${\mathrm{MS}}_{2}$ in Figures 5 and 6, the solid components of ${\mathrm{MS}}_{2}$ were considered to be rigid due to the huge impedance contrast between air and solid. Only the pressure acoustics module was used to perform the calculation. The solid boundaries of ${\mathrm{MS}}_{2}$ unit cells were set as the sound hard boundary to reduce the computational cost. The sound velocity and density were set as $c_{w}=1500\;m\;s^{-1}$ and $\rho_{w}=1000\;\mathrm{kg}\;m^{-3}$ for water and $c_{a}=343\;m\;s^{-1}$ and $\rho_{a}=1.21\;\mathrm{kg}\;m^{-3}$ for air, respectively. The material of solid metasurface was epoxy resin with density $\rho_{m}=1140\;\mathrm{kg}\;m^{-3}$, Young's modulus $E_{m}=3\;\mathrm{GPa},$ and Poisson's ratio µ_m_ = 0.41. When viscosity is considered, Young's modulus of solid material was set as $E_{m}^{\prime }=\left(1+0.05i\right)E_{m}$.

### Experimental Apparatus

The samples of hybrid metasurfaces were fabricated by 3D printing technology. ${\mathrm{MS}}_{1}$ included 24 identical unit cells along the *y*‐axis and was stretched by 520 mm along the *x*‐axis. ${\mathrm{MS}}_{2}$ was made of 24 × 24 unit cells. The water‐to‐air acoustic experimental setup is shown in Figure 1B. An underwater transducer with 10 cm in diameter (Model T313, Neptune Sonar Limited) to emit sound waves was placed on the bottom of the water tank (dimensions 1600 mm × 1500 mm × 800 mm) equipped with sponge and rubber wedges. ${\mathrm{MS}}_{1}$ was assembled in a metal framework and floating at the water–air interface. ${\mathrm{MS}}_{2}$ was placed on top of ${\mathrm{MS}}_{1}$ with about 4.2 mm air gap. A 1/4 inch microphone (Type 4939, Brüel & Kjær) was fixed on the scanning platform to measure the sound fields in the air. The experimental setup of the vibration test for ${\mathrm{MS}}_{1}$ is shown in Figure 4A. A flat mirror was fixed on the platform to adjust the path of the laser beam emitted by the laser head. The displacement component *u*_*z*_ was measured by using the PSV scanning vibrometer. The scan area was located in the center of ${\mathrm{MS}}_{1}$, which covered four unit cells along the *y*‐axis and was around 20 cm along the *x*‐axis. For the scanned sound fields in Figures 3, 5, and 6C,F,I, the transmitted amplitudes and phases of 961 scanned points (31 × 31 array) on each plane were collected by the microphone in the air.

### Statistical Analysis

All statistical analyses were performed with MATLAB software. Details of normalization for the collecting data were separately provided in each figure caption. For quantitative analysis of the performance for ${\mathrm{MS}}_{1}$, 21 × 21 sampling points were selected on the *xy*‐plane. The quadratic mean ratio of measured results with and without ${\mathrm{MS}}_{1}$ was calculated by Equation (10) and presented in Figure 3A. The maximum, minimum as well as data at each sampling point are provided in Note S6, Supporting Information.

## Conflict of Interest

The authors declare no conflict of interest.

## Author Contributions

H.T.Z., Y.‐F.W., and Y.‐S.W. designed the research and conceived the origin idea. Y.F.W. and Y.S.W. supervised the research. H.T.Z. formulated and accomplished all optimizations and designs of metasurfaces. H.T.Z., S.C.Z., and T.Z. performed the experiments. H.T.Z., T.Y.Z., Y.F.W., and Y.S.W. analyzed data. H.T.Z. wrote the paper. All authors discussed the results and commented to the manuscript. Y.F.W. and Y.S.W. revised the paper.

## Supporting information

Supporting Information

Supplemental Movie 1

Supplemental Movie 2

Supplemental Movie 3

Supplemental Movie 4

## Data Availability

The data that support the findings of this study are available from the corresponding author upon reasonable request.
