# Supplementary material for: Hybrid Metasurfaces for Perfect Transmission and Customized Manipulation of Sound Across Water–Air Interface
Source: Adv Sci (Weinh). 2023 Apr 20;10(19):2207181. doi: 10.1002/advs.202207181 (PMC10323646; doi:10.1002/advs.202207181)
Supplement: Supplementary file 1 — Supporting Information [file ADVS-10-2207181-s002.pdf]

ADVANCED SCIENCE
Open Access

Supporting Information

for *Adv. Sci.*, DOI 10.1002/advs.202207181

Hybrid Metasurfaces for Perfect Transmission and Customized Manipulation of Sound Across Water–Air Interface

Hong-Tao Zhou, Shao-Cong Zhang, Tong Zhu, Yu-Ze Tian, Yan-Feng Wang and Yue-Sheng Wang**

Supporting Information

Hybrid metasurfaces for perfect transmission and customized manipulation of sound across water-air interface

Hong-Tao Zhou, Shao-Cong Zhang, Tong Zhu, Yu-Ze Tian,
Yan-Feng Wang, and Yue-Sheng Wang**

H.-T. Zhou, S.-C. Zhang, T. Zhu, Y.-Z. Tian, Y.-F. Wang, Y.-S. Wang
Department of Mechanics
School of Mechanical Engineering
Tianjin University
Tianjin, 300350, China
Email Address: wangyanfeng@tju.edu.cn (Y.-F. Wang); yswang@tju.edu.cn (Y.-S. Wang)

Y.-S. Wang
Institute of Engineering Mechanics
Beijing Jiaotong University
Beijing, 100044, China

Note S1. Analytical derivations of sound transfer matrices T and Φ for the hybrid metasurfaces

Figure S1 illustrates the schematic diagram of the reflection and transmission across water-air interface through the hybrid metasurfaces. One dimensional case with normal incidence from water to air is considered. Under the plane wave assumption, the total sound pressures in each layer of host medium can be expressed as

$$\begin{bmatrix} P_w \\ P_{m_1} \\ P_{m_{12}} \\ P_{m_2} \\ P_a \end{bmatrix} = \begin{bmatrix} p_i e^{-ik_w z} + p_r e^{ik_w z} \\ p_{i_1} e^{-ik_{m_1} z} + p_{r_1} e^{ik_{m1} z} \\ p_{i_{12}} e^{-ik_a (z-d_{m_1})} + p_{r_{12}} e^{ik_a (z-d_{m_1})} \\ p_{i_2} e^{-ik_{m_2}(z-d_{m_1}-d_{m_{12}})} + p_{r_2} e^{ik_{m2}(z-d_{m_1}-d_{m_{12}})} \\ p_t e^{-ik_a (z-d_{m_1}-d_{m_{12}}-d_{m_2})} \end{bmatrix}, \tag{S1}$$

where k defines the propagating wavenumber in each host medium; and the subscripts w, a, m_1, m_2 and m_{12} represent the water, the air, MS_1, MS_2 and the air gap, respectively. Accordingly, the associated velocity fields can be obtained as

$$\begin{bmatrix} v_w \\ v_{m_1} \\ v_{m_{12}} \\ v_{m_2} \\ v_a \end{bmatrix} = \begin{bmatrix} \frac{p_i}{Z_w} e^{-ik_w z} - \frac{p_r}{Z_w} e^{ik_w z} \\ \frac{p_{i_1}}{Z_{m_1}} e^{-ik_{m_1} z} - \frac{p_{r_1}}{Z_{m_1}} e^{ik_{m1} z} \\ \frac{p_{i_{12}}}{Z_a} e^{-ik_a (z-d_{m_1})} - \frac{p_{r_{12}}}{Z_a} e^{ik_a (z-d_{m_1})} \\ \frac{p_{i_2}}{Z_{m_2}} e^{-ik_{m_2}(z-d_{m_1}-d_{m_{12}})} - \frac{p_{r_2}}{Z_{m_2}} e^{ik_{m2}(z-d_{m_1}-d_{m_{12}})} \\ \frac{p_t}{Z_a} e^{-ik_a (z-d_{m_1}-d_{m_{12}}-d_{m_2})} \end{bmatrix}, \tag{S2}$$

where Z indicates the acoustic characteristic impedance. Furthermore, the continuous conditions of sound pressure and velocity at the interfaces between host medium are written as

$$\begin{bmatrix} P_w|_{z=0} \\ P_{m_1}|_{z=d_{m_1}} \\ P_{m_{12}}|_{z=d_{m_1}+d_{m_{12}}} \\ P_{m_2}|_{z=d_{m_1}+d_{m_{12}}+d_{m_2}} \\ v_w|_{z=0} \\ v_{m_1}|_{z=d_{m_1}} \\ v_{m_{12}}|_{z=d_{m_1}+d_{m_{12}}} \\ v_{m_2}|_{z=d_{m_1}+d_{m_{12}}+d_{m_2}} \end{bmatrix} = \begin{bmatrix} P_{m_1}|_{z=0} \\ P_{m_{12}}|_{z=d_{m_1}} \\ P_{m_2}|_{z=d_{m_1}+d_{m_{12}}} \\ P_a|_{z=d_{m_1}+d_{m_{12}}+d_{m_2}} \\ v_{m_1}|_{z=0} \\ v_{m_{12}}|_{z=d_{m_1}} \\ v_{m_2}|_{z=d_{m_1}+d_{m_{12}}} \\ v_a|_{z=d_{m_1}+d_{m_{12}}+d_{m_2}} \end{bmatrix}. \tag{S3}$$

Then, the transfer relationships between sound pressure amplitudes in each layer of host medium can be established by solving Equation (S3), and we have

$$\begin{bmatrix} p_{i_1} \\ p_{r_1} \end{bmatrix} = \mathbf{T_0} \begin{bmatrix} p_i \\ p_r \end{bmatrix} = \frac{1}{2} \begin{bmatrix} 1 + Z_{m_1}/Z_w & 1 - Z_{m_1}/Z_w \\ 1 - Z_{m_1}/Z_w & 1 + Z_{m_1}/Z_w \end{bmatrix} \begin{bmatrix} p_i \\ p_r \end{bmatrix}, \tag{S4}$$

$$\begin{bmatrix} p_{i_{12}} \\ p_{r_{12}} \end{bmatrix} = \mathbf{T_1} \begin{bmatrix} p_{i_1} \\ p_{r_1} \end{bmatrix} = \frac{1}{2} \begin{bmatrix} (1 + Z_a/Z_{m_1})e^{-ik_{m_1}d_{m_1}} & (1 - Z_a/Z_{m_1})e^{ik_{m_1}d_{m_1}} \\ (1 - Z_a/Z_{m_1})e^{-ik_{m_1}d_{m_1}} & (1 + Z_a/Z_{m_1})e^{ik_{m_1}d_{m_1}} \end{bmatrix} \begin{bmatrix} p_{i_1} \\ p_{r_1} \end{bmatrix}, \tag{S5}$$

$$\begin{bmatrix} p_{i_2} \\ p_{r_2} \end{bmatrix} = \mathbf{T_2} \begin{bmatrix} p_{i_{12}} \\ p_{r_{12}} \end{bmatrix} = \frac{1}{2} \begin{bmatrix} (1 + Z_{m_2}/Z_a)e^{-ik_a d_{m_{12}}} & (1 - Z_{m_2}/Z_a)e^{ik_a d_{m_{12}}} \\ (1 - Z_{m_2}/Z_a)e^{-ik_a d_{m_{12}}} & (1 + Z_{m_2}/Z_a)e^{ik_a d_{m_{12}}} \end{bmatrix} \begin{bmatrix} p_{i_{12}} \\ p_{r_{12}} \end{bmatrix}, \tag{S6}$$

$$\begin{bmatrix} p_a \\ 0 \end{bmatrix} = \mathbf{T_3} \begin{bmatrix} p_{i_2} \\ p_{r_2} \end{bmatrix} = \frac{1}{2} \begin{bmatrix} (1 + Z_a/Z_{m_2})e^{-ik_{m_2}d_{m_2}} & (1 - Z_a/Z_{m_2})e^{ik_{m_2}d_{m_2}} \\ (1 - Z_a/Z_{m_2})e^{-ik_{m_2}d_{m_2}} & (1 + Z_a/Z_{m_2})e^{ik_{m_2}d_{m_2}} \end{bmatrix} \begin{bmatrix} p_{i_2} \\ p_{r_2} \end{bmatrix}. \tag{S7}$$

Based on Equation (S4)-(S7), the pressure amplitude relationships across the hybrid metasurfaces can be solved as

$$\begin{bmatrix} p_a \\ 0 \end{bmatrix} = \mathbf{T_3 T_2 T_1 T_0} \begin{bmatrix} p_i \\ p_r \end{bmatrix} \overset{\text{def}}{=} \mathbf{\Phi T} \begin{bmatrix} p_i \\ p_r \end{bmatrix}, \tag{S8}$$

where $\mathbf{\Phi} = \mathbf{T_3 T_2}$ and $\mathbf{T} = \mathbf{T_1 T_0}$. It is noted that $p_a = p_{i_{12}} e^{-i(k_a d_{m_{12}} + k_{m_2} d_{m_2})}$ when $Z_{m_2} = Z_a$. This means that $\mathrm{MS_2}$ is mainly used for the phase modulation since a unitary transmission from air to air can be obtained due to the identical impedance of $\mathrm{MS_2}$ and air. Here, we consider $Z_{m_2} = Z_a$ during the design of $\mathrm{MS_2}$ for simplicity. Then $\mathbf{\Phi}$ degenerates into a unitary matrix, and $\mathbf{T} = \mathbf{T_1 T_0}$ mainly represents the enhanced transmission from water to air by $\mathrm{MS_1}$. And the elements in $\mathbf{T}$ and $\mathbf{\Phi}$ can be solved based on Equation (S4)-(S7).

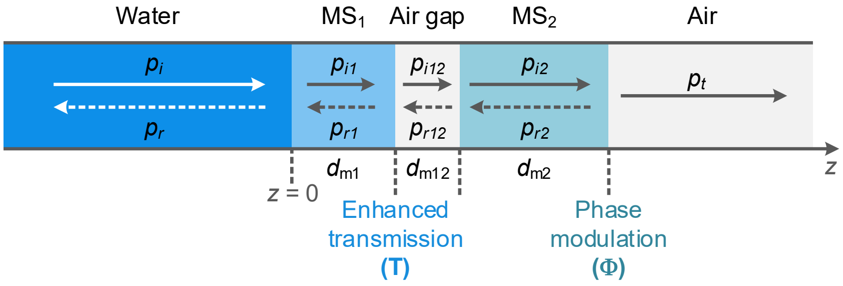

Figure S1: Schematic diagram of the reflection and transmission across water-air interface through hybrid metasurfaces. Sound waves are incident from the water on the left. p_i and p_r represent the complex sound pressure amplitudes of incident and reflected waves in the water; p_t indicates the complex sound pressure amplitude of transmitted wave in the air. $\mathrm{MS_1}$ and $\mathrm{MS_2}$ are treated as the homogeneous medium with thickness d_{m_1} and d_{m_2}, respectively; and p_{i_1}, p_{r_1} and p_{i_2}, p_{r_2} represent the complex pressure amplitudes of sound waves propagating in $\mathrm{MS_1}$ and $\mathrm{MS_2}$ along $\pm x$ axis directions, respectively. $\mathrm{MS_1}$ and $\mathrm{MS_2}$ are separated by an air gap with the thickness of $d_{m_{12}}$, and $p_{i_{12}}$ and $p_{r_{12}}$ indicate the complex sound pressure amplitudes of incident and reflected waves in the air gap.

Note S2. Retrieving method for reflected/transmitted sound intensity coefficients of MS_1 and phase/transmission of MS_2

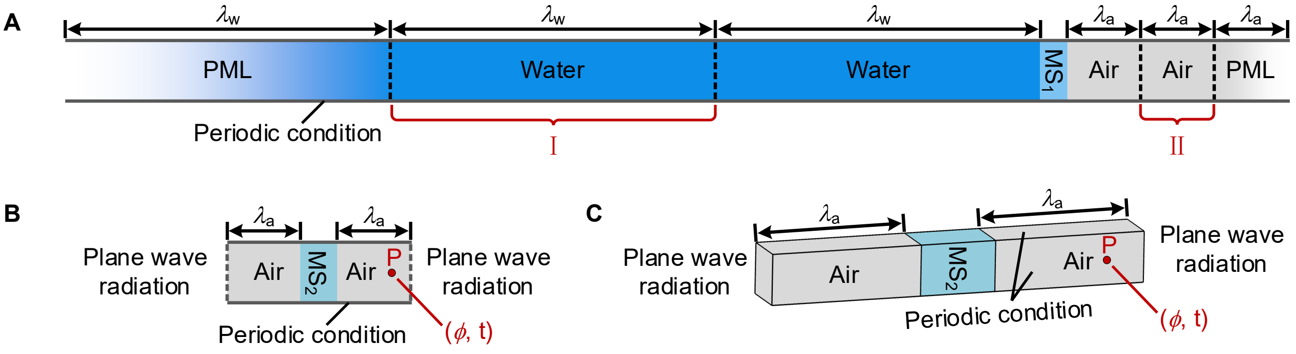

Figure S2: Illustration of retrieving method based on the finite element calculation. Panel (A) shows the retrieving method for reflection/ transmission sound intensity coefficients of MS_1 unit. I and II indicate the observation areas for retrieving reflection/ transmission sound intensity, respectively. Panels (B) and (C) present the retrieving method for the transmitted phase and transmission of 2D and 3D MS_2 units, respectively. P represents the observation point for retrieving the transmitted phase and transmission.

Figure S2(A) illustrates the retrieving method for reflected/transmitted sound intensity coefficients (I_R and I_T) of MS_1 unit. The 2D plane strain approximation is considered for the solid MS_1 unit. Numerical calculations are conducted by using the finite element software COMSOL. Periodic condition is applied on the water-to-air waveguide. Perfectly matched layers at the ends of the water and air domains are used to eliminate boundary reflections. To avoid the influence of evanescent waves, the observation areas of the incident and transmitted ends (I and II) are chosen to be a wavelength away from the surface of MS_1 unit. Then I_R and I_T are calculated by

$$I_R = \frac{\int_I \left|\frac{1}{2}\,\mathrm{Re}\left(p_r v_r^*\right)\right| dS_I/S_I}{\int_I \left|\frac{1}{2}\,\mathrm{Re}\left(p_b v_b^*\right)\right| dS_I/S_I},\; I_T = \frac{\int_{II} \left|\frac{1}{2}\,\mathrm{Re}\left(p_t v_t^*\right)\right| dS_{II}/S_{II}}{\int_I \left|\frac{1}{2}\,\mathrm{Re}\left(p_b v_b^*\right)\right| dS_I/S_I}, \tag{S9}$$

where p_b, p_r and p_t indicate the incident background sound pressure, reflected sound pressure in the observation region I, and transmitted sound pressure in the observation region II; v_b, v_r and v_t are the corresponding velocity along the normal (z axis) direction; S_I and S_{II} are the areas of regions I and II; and $*$ represents the conjugation. I_T' is also calculated by Equation (S9) when the viscosity of MS_1 unit is considered.

In terms of the inverse design of MS_2 units, we adopt a two-step optimization from 2D to 3D. Figure S2(B) and S2(C) illustrate the 2D and 3D schematics of retrieving method for the phase shift and transmission (ϕ and t) of MS_2 units, respectively. The air-to-air waveguide is employed under periodic condition. For simplicity, the plane wave radiation condition is applied to avoid boundary reflections. The

observation point P is chosen to be $0.75\lambda_a$ away from the surface of MS_2 unit. ϕ and t can be obtained by taking the phase angle and amplitude of the transmitted sound pressure at the observation point P.

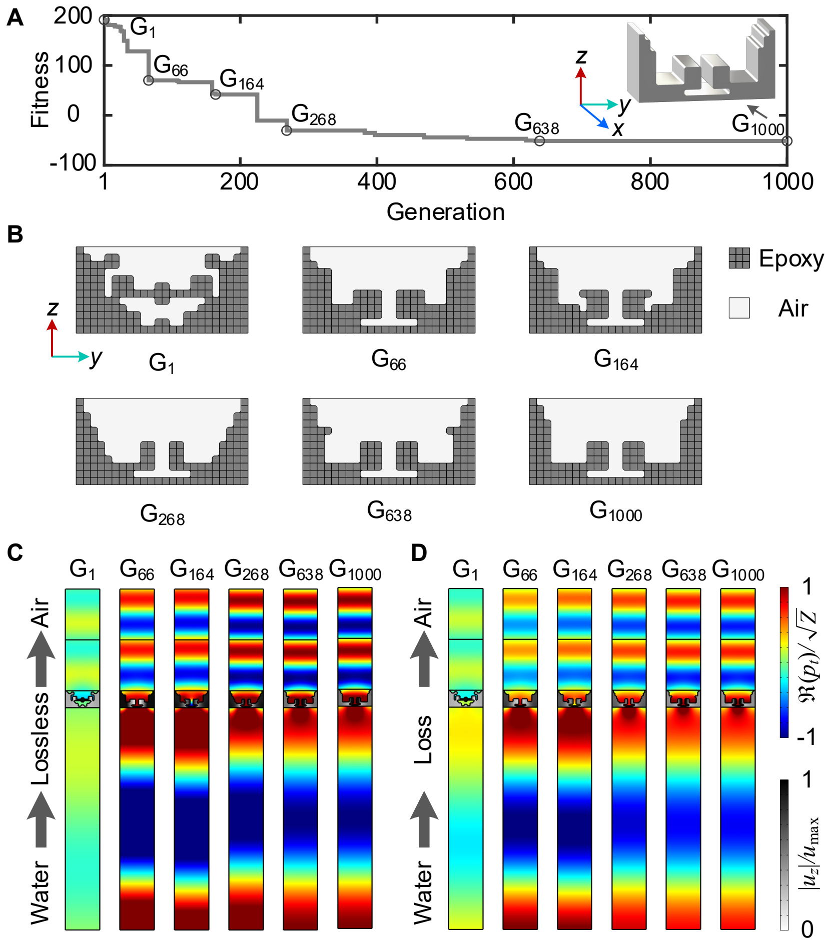

Figure S3: Inverse optimization design process of MS_1 unit. Panel (A) illustrates the evolution history of optimized MS_1 unit. The inset on the right in the upper right corner illustrates the optimized MS_1 unit. The corresponding topological configurations at marked generations (G_1, G_{66}, G_{164}, G_{268}, G_{638}, and G_{1000}) are presented in panel (B). The corresponding water-air sound transmission calculation of these MS_1 units without and with losses are shown in panels (C) and (D), respectively. The simulated results show the total sound pressure fields normalized by the root of water or air acoustic impedance and displacement fields along z-axis direction of MS_1 units.

Note S3. Inverse design process of hybrid metasurfaces

For the inverse design of hybrid metasurfaces, the MS_1 and MS_2 are separately optimized due to the decoupled modulation of sound. The evolution history of optimized MS_1 unit is illustrated in Figure S3(A). It can be seen that the fitness drops from 191.4810 in the first generation to -51.9517 in the

last generation. This means that the optimized MS_1 unit achieves a transition from nearly total reflection to full transmission in the lossless case. When loss is introduced, the optimized MS_1 can also achieve over 50% sound energy transmission across water-air interface.

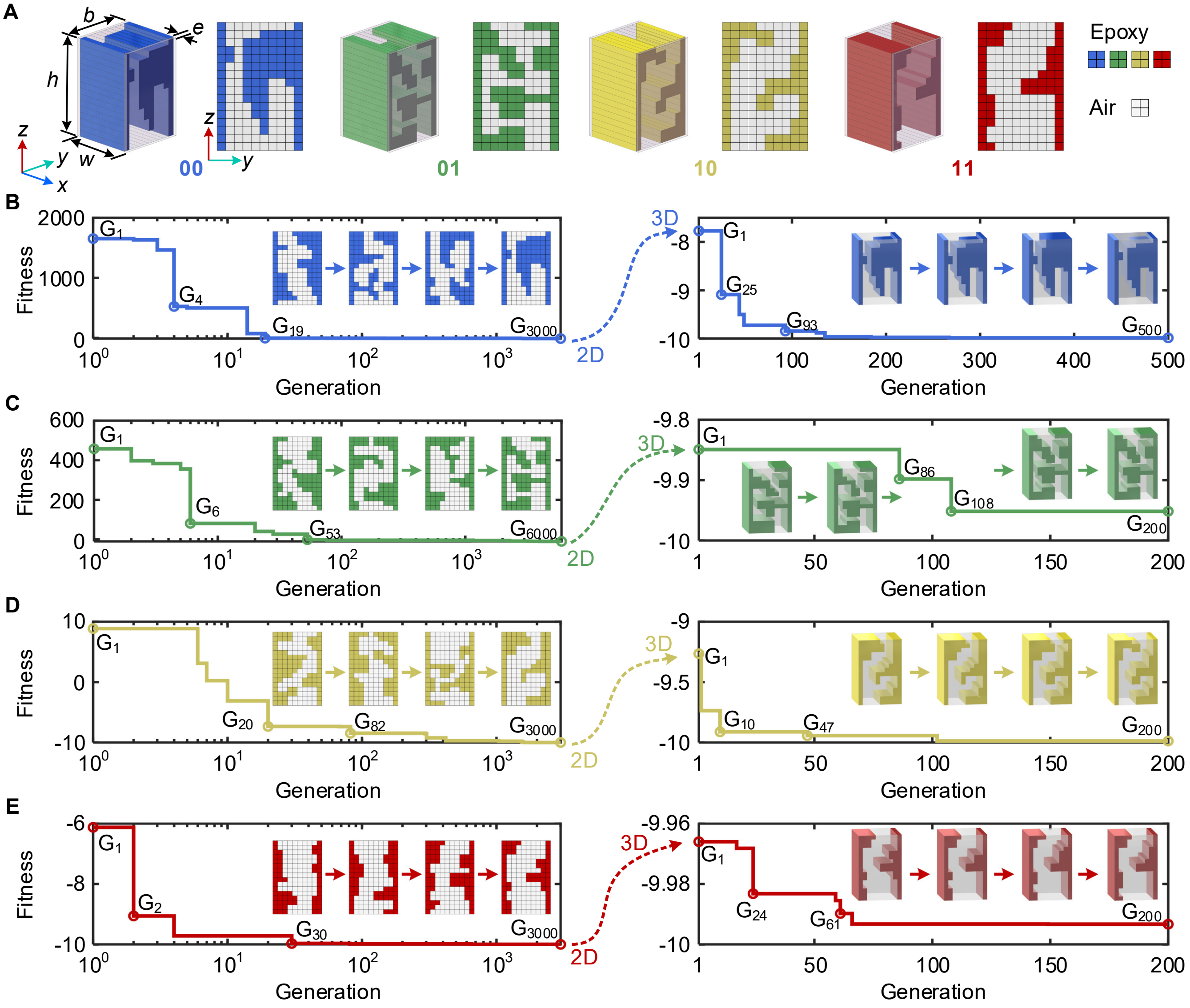

Figure S4: Inverse optimization design process of MS_2 units. Panel (A) presents the optimized 4 coding MS_2 units (00, 01, 10 and 11). The 3D units have the dimensions of $b \times w \times h = \lambda_a/3 \times \lambda_a/3 \times \lambda_a/2$, which are obtained by stretching the 2D optimized MS_2 units by the thickness of $w-2e$, then adding plates with thickness of e on both sides. Panels (B)-(E) illustrate the evolution history of 4 coding MS_2 units, respectively, where the 2D and 3D optimization processes of these units are presented on the left and right, respectively. The insets on the upper right corner illustrates the corresponding topological configurations at marked generations.

To clearly show the evolutionary process, the topological configurations of MS_1 units at the marked generations in Figure S3(A) are presented in Figure S3(B). It is observed from Figure S3(B) that the optimization procedure can quickly capture the fundamental topological features with resonant masses and cavities, and then make the MS_1 units evolve towards the target. It is noted that we perform the smooth processing to the corners inside MS_1 units by chamfering. The chamfer radius is set as 1/3 of the height of a pixel, namely $d_{m_1}/36$. This smooth geometrical operation can significantly improve mesh convergence especially for the finite element calculation with fluid-solid interactions. Figure S3(C) and S3(D) show the corresponding water-to-air sound transmission calculation of these optimized units without and with losses, respectively. As the optimization proceeds, the transmitted sound pressure amplitude by MS_1 unit with and without loss gradually increases with the number of iterations. Therefore, the MS_1 unit with enhanced sound transmission across the water-air interface is successfully designed by finding the minimum fitness function.

Figure S4(A) shows the 3D topological configurations of 4 optimized MS_2 units (00, 01, 10 and 11), and the corresponding 2D designed layouts are presented on the right, which is divided by $N_x \times N_y = 10 \times 16$ pixels. By stretching and adding the plates on both sides, the 2D MS_2 unit can be transformed into 3D one with $b \times w \times h = \lambda_a/3 \times \lambda_a/3 \times \lambda_a/2$ ($d_{m_2} = h$), where the thickness of plate (e) is set as $w/20$. Figure S4(B)-S4(E) present the evolution history of 4 encoded MS_2 units from 2D to 3D, respectively. The insets in each panel illustrate the topological configurations of MS_2 units at the marked generations. It can be seen that the fitness functions decrease rapidly during the 2D optimization, which suggests that appropriate topology configurations are found to provide the desired phase shift and unitary transmission. The 3D optimization starts with the 2D optimized results, and after a few iterations, the optimized MS_2 units are found.

Note S4. Effective acoustic impedance and wavenumber of optimized MS_1

Figure S5 illustrates the retrieving method for effective acoustic impedance and wavenumber of optimized MS_1 unit. The MS_1 is regarded as a homogeneous medium with effective acoustic impedance $Z_{m_1}$and wavenumber k_{m_1}. The periodic condition boundary is imposed on the both sides of MS_1 unit. Perfect matching layer is applied to avoid the boundary reflections. When the uniform plane wave incidents on the MS_1 unit from water, the total sound pressures in the water (P_w), MS_1 (P_{m_1}) and air (P_a) can be expressed as

$$\begin{bmatrix} P_w \\ P_{m_1} \\ P_a \end{bmatrix} = \begin{bmatrix} Ae^{-ik_w z} + Be^{ik_w z} \\ Ce^{-ik_{m1} z} + De^{ik_{m1} z} \\ Ee^{-ik_a(z-d_{m_1})} + Fe^{ik_a(z-d_{m_1})} \end{bmatrix}, \tag{S10}$$

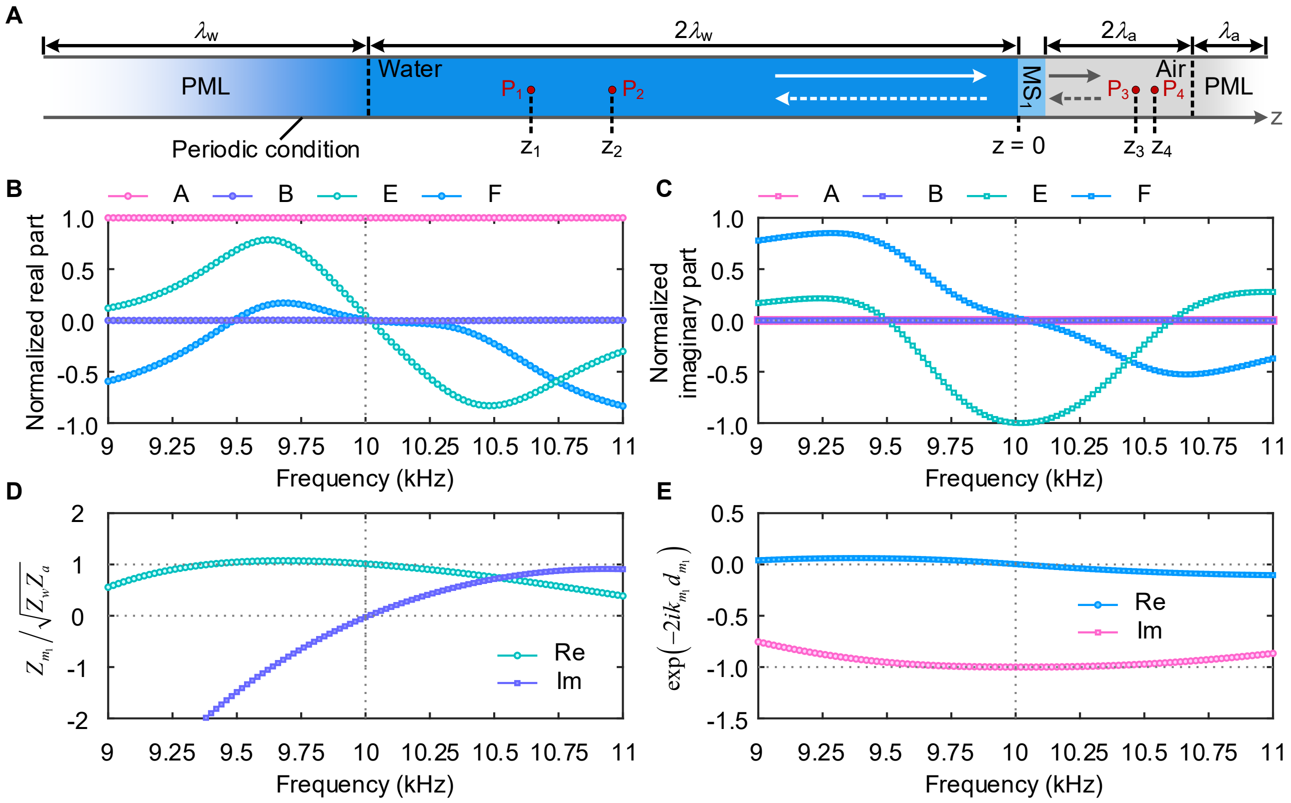

Figure S5: Retrieving method for effective acoustic impedance and wavenumber of optimized MS_1 unit. Panel (A) illustrates the schematic of the method based on the standard 4-microphone for acoustic experiments to retrieve the complex sound pressure amplitudes in COMSOL software. Sound waves are incident from the water on the left. P_1, P_2, P_3 and P_4 are the observation points for extracting sound pressure. The coordinates of them are z_1, z_2, z_3 and z_4 along the z axis direction, respectively. Periodic boundary condition is applied to the both sides. Perfectly matched layer (PML) is used to eliminate boundary reflections. The normalized real and imaginary parts of complex sound pressure amplitudes in water and air domains as a function of frequency for the optimized MS_1 unit by retrieving in the COMSOL software are plotted panels (B) and (C), respectively. Panels (D) and (E) present the variations of Z_{m_1} and $\exp(2ik_{m_1}d_{m_1})$ with the incident frequency calculated by Equation (S12). At the peak frequency 10 kHz, the water-to-air full-transmission conditions for MS_1 given by quarter-wave impedance-matched theory are well satisfied.

where A, B; C, D; and E, F indicate the complex sound pressure amplitudes along the $\pm z$-axis in water, MS_1 and air. Accordingly, the associated velocity fields can be calculated as

$$\begin{bmatrix} v_w \\ v_{m_1} \\ v_a \end{bmatrix} = \begin{bmatrix} \dfrac{A}{Z_w} e^{-ik_w z} - \dfrac{B}{Z_w} e^{ik_w z} \\ \dfrac{C}{Z_{m_1}} e^{-ik_{m_1} z} - \dfrac{D}{Z_{m_1}} e^{ik_{m1} z} \\ \dfrac{E}{Z_a} e^{-ik_a(z-d_{m_1})} - \dfrac{E}{Z_a} e^{ik_a(z-d_{m_1})} \end{bmatrix}, \tag{S11}$$

Furthermore, the continuous conditions of sound pressure and velocity at the interfaces between host medium are written as

$$\begin{bmatrix} P_w|_{z=0} \\ P_{m_1}|_{z=d_{m_1}} \\ v_w|_{z=0} \\ v_{m_1}|_{z=d_{m_1}} \end{bmatrix} = \begin{bmatrix} P_{m_1}|_{z=0} \\ P_a|_{z=d_{m_1}} \\ v_{m_1}|_{z=0} \\ v_a|_{z=d_{m_1}} \end{bmatrix}. \tag{S12}$$

By substituting Equation (S10) and (S11) into Equation (S12), we can establish the relationships between effective acoustic impedance, wavenumber and the complex sound pressure amplitudes as

$$\begin{bmatrix} Z_{m_1} \\ \exp\left(2ik_{m_1}d_{m_1}\right) \end{bmatrix} = \begin{bmatrix} Z_w Z_a\sqrt{\dfrac{(A+B)^2-(E+F)^2}{\left(Z_a\right)^2(A-B)^2-\left(Z_w\right)^2(E-F)^2}} \\ \dfrac{\left[Z_w(A+B)+Z_{m_1}(A-B)\right]\left[Z_a(E+F)-Z_{m_1}(E-F)\right]}{\left[Z_w(A+B)-Z_{m_1}(A-B)\right]\left[Z_a(E+F)+Z_{m_1}(E-F)\right]} \end{bmatrix}. \tag{S13}$$

For the ease of implementation, we will employ the 4-microphone-based approach to calculate the complex sound pressure amplitudes in COMSOL software, as shown in Figure S5 (A). The 4 selected sample points $P_1,\ P_2,\ P_3,\ P_4$ are located on the central axis of the z axis direction coordinates $z_1,\ z_2,\ z_3$ and z_4. The extracted sound pressures $P_{t_1},\ P_{t_2},\ P_{t_3},\ P_{t_4}$ at the 4 points satisfy the condition

$$\begin{bmatrix} P_{t_1} \\ P_{t_2} \\ P_{t_3} \\ P_{t_4} \end{bmatrix} = \begin{bmatrix} Ae^{-ik_w z_1}+Be^{ik_w z_1} \\ Ae^{-ik_w z_2}+Be^{ik_w z_2} \\ Ee^{-ik_a\left(z_3-d_{m_1}\right)}+Fe^{ik_a\left(z_3-d_{m_1}\right)} \\ Ee^{-ik_a\left(z_4-d_{m_1}\right)}+Fe^{ik_a\left(z_4-d_{m_1}\right)} \end{bmatrix}. \tag{S14}$$

Therefore, the complex sound pressure amplitudes in water and air domains can be calculated as

$$\begin{bmatrix} A \\ B \\ E \\ F \end{bmatrix} = \begin{bmatrix} \dfrac{P_{t_1}e^{-ik_w z_1}-P_{t_2}e^{-ik_w z_2}}{e^{-2ik_w z_1}-e^{-2ik_w z_2}} \\ \dfrac{P_{t_1}e^{ik_w z_1}-P_{t_2}e^{ik_w z_2}}{e^{2ik_w z_1}-e^{2ik_w z_2}} \\ \dfrac{P_{t_3}e^{-ik_a z_3}-P_{t_4}e^{-ik_a z_4}}{e^{-2ik_a\left(z_3-d_{m_1}\right)}-e^{-2ik_a\left(z_4-d_{m_1}\right)}} \\ \dfrac{P_{t_3}e^{ik_a\left(z_3-d_{m_1}\right)}-P_{t_4}e^{ik_a\left(z_4-d_{m_1}\right)}}{e^{2ik_a\left(z_3-d_{m_1}\right)}-e^{2ik_a\left(z_4-d_{m_1}\right)}} \end{bmatrix}. \tag{S15}$$

Combining Equation (S13) and (S15), we finally obtain the effective acoustic impedance and wavenumber of MS_1.

Figures S5(B) and S5(C) show the normalized real and imaginary parts of complex sound pressure amplitudes in water and air domains as a function of frequency for the optimized MS_1 unit retrieved in the

COMSOL software, respectively, where we have $z_2 - z_1 = \lambda_w/4$, $z_4 - z_3 = \lambda_a/4$, $z_1 = -3\lambda_w/2$, and $z_3 = d_{m_1} + 3\lambda_a/2$ in the calculation. It can be seen that $A = 1$ and $F \approx 0$ hold for all selected frequencies since the uniform plane wave background field and the perfectly matched layer are applied. When the incident frequency approaches 10 kHz, the reflected amplitude B decreases to 0 while the normalized transmitted amplitude E increases to -i (obtained by multiplying by $\sqrt{Z_w/Z_a}$). A distinguished phase difference of $\pi/2$ between A and E at the peak frequency can be observed as expected, showing a good agreement with the theoretical analysis in the main text. Furthermore, Figure S5(D) and S5(E) present Z_{m_1} and $\exp(2ik_{m_1}d_{m_1})$ retrieved by Equation (S13), respectively. $\Re(Z_{m_1}) \approx \sqrt{Z_w Z_a}$ and $\Im(Z_{m_1}) \approx 0$ are observed at the peak frequency. Meanwhile, $\exp(2ik_{m_1}d_{m_1}) \approx \exp(i\pi) = -1$. These results suggest that the cross-media full-transmission conditions for MS_1 given by quarter-wave impedance-matched theory are well satisfied, thus validating the practicability of proposed concept.

Note S5. Weak coupling effect between hybrid metasurfaces

Figure S6(A) shows the variation of water-to-air sound transmission with the distance $d_{m_{12}}$ between MS_1 and MS_2, and the corresponding phase shifts as a function of $d_{m_{12}}$ are shown in Figure S6(B). It can be seen that the normalized sound pressure transmission of the 4 MS_2 units hybrid with MS_1 units can also keep at about 1 even for the rather narrow distance $d_{m_{12}} = \lambda_a/100$, and the phase shifts also maintain near the desired values. These results indicate the weak coupling effect between hybrid metasurfaces, verifying the feasibility of the proposed concept for enhanced transmission and phase manipulation across water-air interface.

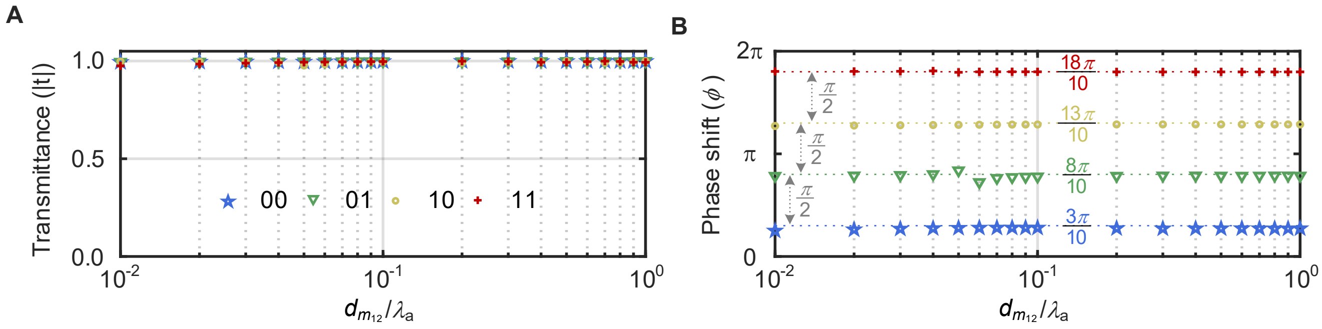

Figure S6: Weak coupling effect between hybrid metasurfaces. Panels (A) and (B) presents the water-to-air sound transmission and transmitted phase shifts of the hybrid MS_1 and MS_2 units, respectively, where the symbols of pentagram, triangle, circle and plus sign represent the hybrid units 00, 01, 10 and 11, respectively.

Note S6. Measurement on sound transmission by $\mathrm{MS_1}$ and the bare water-air interface

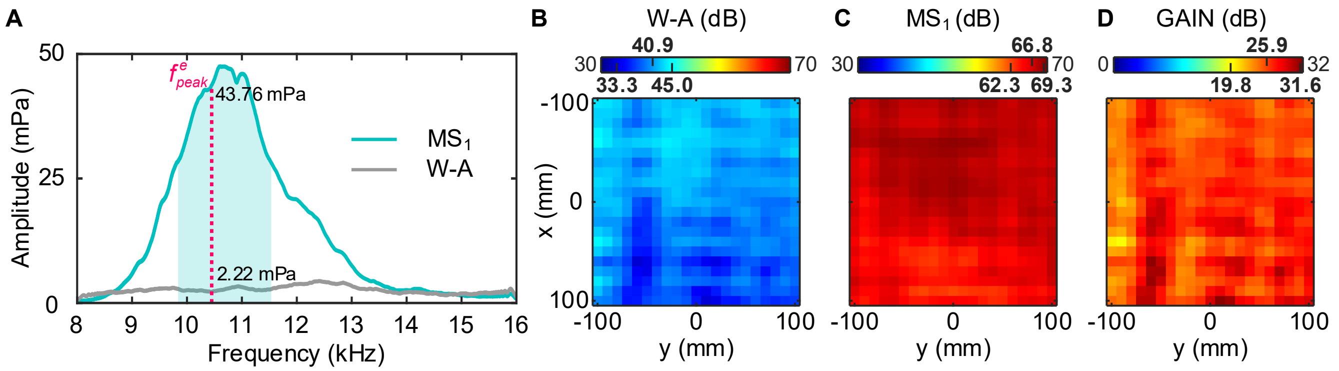

Figure S7: Experimental measured results of water-to-air sound transmission through the $\mathrm{MS_1}$ and the bare water-air interface. Panel (A) shows the average sound pressure amplitudes transmitted to air through the optimized $\mathrm{MS_1}$ and the bare water-air interface as a function of frequency. The peak frequency is marked as $f^e_{peak} = 10.45$ kHz. Panels (B) and (C) present the measured sound transmission amplitudes on the $x - y$ plane (21 × 21 samples points) through the bare water-air interface and $\mathrm{MS_1}$ at the peak frequency, respectively. The minimal, maximal, and quadratic mean amplitudes of 40.9 dB (2.22 mPa) and 66.8 dB (43.76 mPa) for the both are marked in the corresponding color bars, respectively. The corresponding sound transmission enhancements at each measured point are presented in panel (D), where the minimal, maximal, and average enhancements of 25.9 dB at the peak frequency are marked in the color bar.

Figure S7(A) shows the experimental measured sound transmission amplitudes through the optimized $\mathrm{MS_1}$ and the bare water-air interface as a function of frequency, which are obtained by calculating the quadratic mean of 21×21 samples points on the $x - y$ plane. It is obviously observed that the transmitted sound pressure amplitudes are significantly enhanced over a certain wide frequency range from 9.84 kHz to 11.53 kHz (the shadow area). The measured sound transmission amplitudes at each sample point through the bare water-air interface and $\mathrm{MS_1}$ at the peak frequency are presented in Figure S7(B) and S7(C), respectively. Figure S7(D) further plots the corresponding transmission amplitude enhancement at each measured point. At the peak frequency, the minimal, average, maximal transmitted amplitudes are 33.3 dB, 40.9 dB, and 45.0 dB for the bare water-air interface, respectively, while 62.3 dB, 66.8 dB, and 69.3 dB for the $\mathrm{MS_1}$. Therefore, amplitude enhancement over 20 dB can be observed at almost every point, thus validating the excellent enhanced water-to-air sound transmission performance of $\mathrm{MS_1}$.

Note S7. Wide-angle transmission enhancement of $\mathrm{MS_1}$ under oblique incidences

To demonstrate the wide-angle transmission enhancement of $\mathrm{MS_1}$, we simulate water-to-air sound transmission of $\mathrm{MS_1}$ unit as functions of the incident angle θ_w and the frequency, as shown in

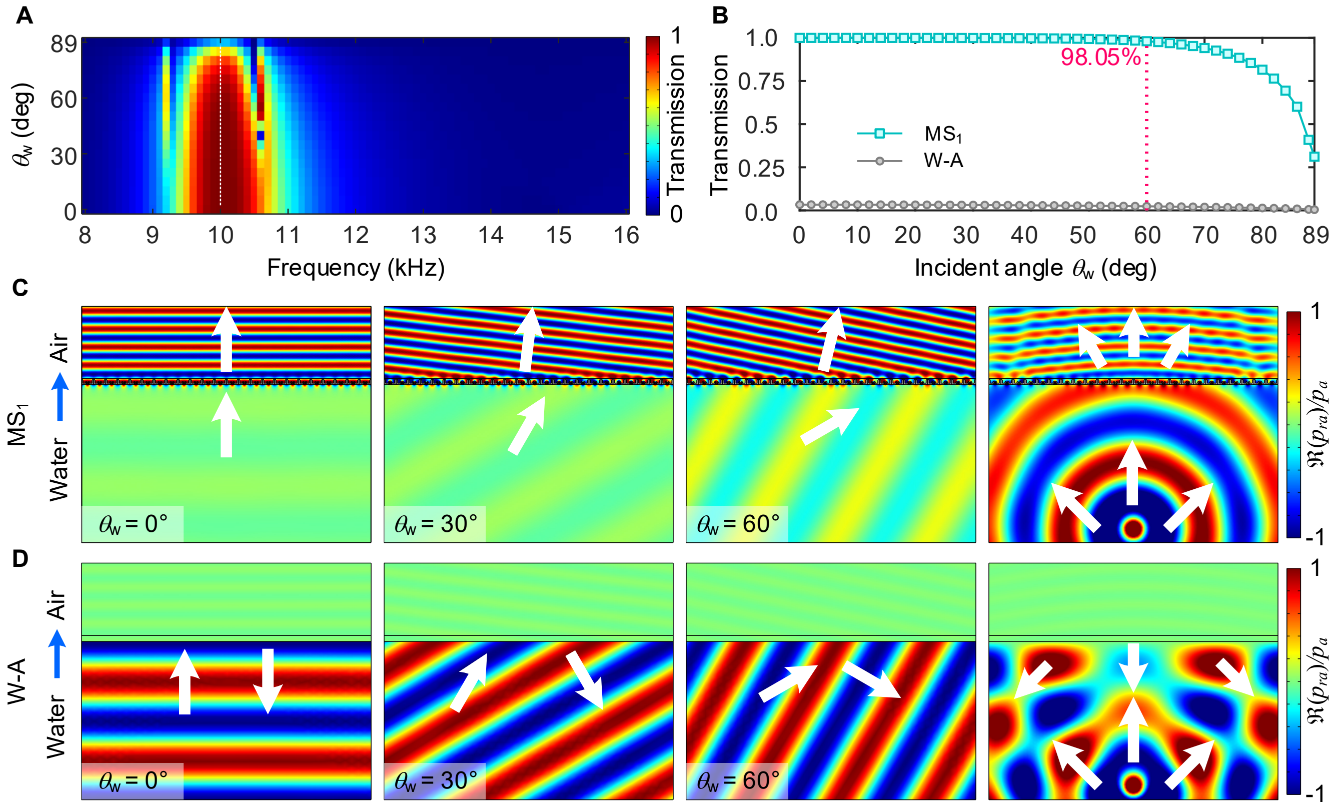

Figure S8: Wide-angle water-to-air sound transmission enhancement performance of optimized MS_1. Panel (A) presents the water-to-air sound pressure transmission of MS_1 as a function of incident angle (from $0°$ to $89°$) and frequency (from 8 kHz to 16 kHz). The variation of water-to-air sound pressure transmission through MS_1 with the incident angle at 10 kHz is plotted in panel (B) (marked by the white dashed in panel (A)). Panel (C) shows the simulated reflected and transmitted sound pressure fields through MS_1 under different incident angles and a point source. The corresponding results through a bare wtaer-to-air interface are presented in panel (D) for comparison. The normalized pressure $p_a = \sqrt{Z_w \cos\theta_a}$ for the water domain and $p_a = \sqrt{Z_a \cos\theta_w}$ for the air domain, where θ_a being the transmission angle in air and θ_w satisfy the Snell's law $k_a \sin\theta_a = k_w \sin\theta_w$.

Figure S8(A). The normalized transmission is calculated by of the square root of the ratio of normal energy intensity of transmission and incidence. It is observed that the optimized MS_1 exhibits a relatively high water-to-air sound transmission coefficient under a wide-frequency and wide-angle incidence range. In addition, it is also noticed that other transmission peak frequencies may appear when the oblique incidence angle is relatively large, which may be attributed to the coupling resonance of the MS_1 with the incident waves. Furthermore, we plot the variations of water-to-air sound transmission through MS_1 and the bare water-air interface with the incident angle θ_w at the peak frequency 10 kHz. It can be seen that the MS_1 can improve the sound pressure transmission from around 3.3% to over 98% within $60°$ oblique

incidence. To clearly show the wide-angle enhanced transmission performance of MS_1 across water-air interface, the full-wave simulated sound pressure fields through MS_1 and the bare water-air interface are presented in Figure S8(C) and S8(D), respectively. The sound waves incident from water can be effectively transmitted to the air with the help of optimized MS_1 for either single-angle oblique incidence or wide-angle point source incidence. Overall, MS_1 exhibits good wide-angle sound transmission enhancement at the water-air interface over a wide frequency range.

Note S8. Measured transmitted sound fields from water to air near the peak frequency

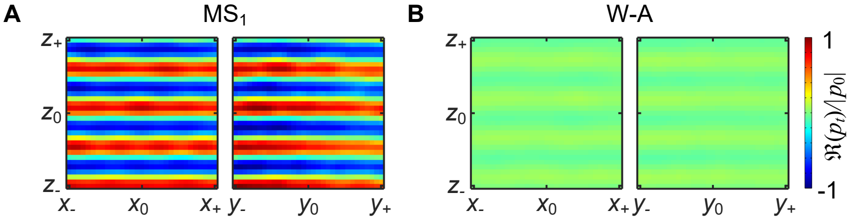

Figure S9: Measured transmitted sound fields from water to air near the peak frequency. Panel (A) presents the measured real parts of transmitted sound fields in $x - z$ and $y - z$ planes from water to air through the MS_1 at 11 kHz. The corresponding measured results for the water-air interface are given in panel (B) for comparison, where $|p_0| = 0.67$ Pa, $z_0 \approx 113.3$ mm, $x_0 = y_0 = 0$ mm, $z_\pm = z_0 \pm 60$ mm, $x_\pm = x_0 \pm 60$ mm and $y_\pm = y_0 \pm 60$ mm.

The measured sound pressure fields transmitted by MS_1 and bare water-air interface at x-z and y-z planes at 11 kHz are shown in Figure S9(A) and S9(B), respectively. Compared with the bare water-air interface, the enhanced uniform plane waves transmitted through the MS_1 can also be clearly observed near the experimental peak frequency.

Note S9. Influence of material parameters on the peak frequency

The offset between the experimental and simulated peak frequencies (10.45 kHz and 10 kHz, respectively) may be attributed to the difference of material parameters between actually fabricated and simulated samples. Here, we investigate the influence of Young's Modulus (E) of solid material on peak frequency shift, as shown in Figure S10. The enhanced amplitude transmission through MS_1 with different Young's modulus are observed without and with losses. When the viscosity in the solid material is not considered, the peak frequency shifts upward with the increase of Young's modulus. However, when the viscosity is considered, the peak frequency is concentrated at 10 kHz for $E_0 < 3$ GPa (the simulated modulus). When $E_0 > 3$ GPa, the peak frequency also shifts upward with Young's modulus

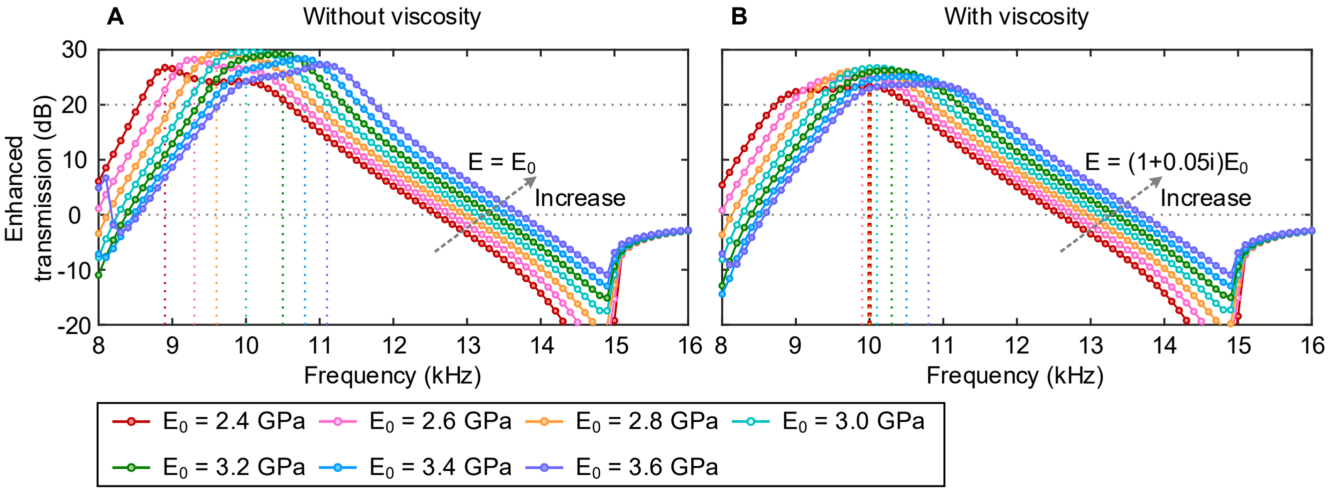

Figure S10: Influence of material parameters on peak frequency of water-to-air sound transmission. Panes (A) presents the enhanced water-to-air sound pressure amplitude transmission as a function of frequency through the MS_1 with different Young's modulus, respectively. The corresponding results through the MS_1 with viscosity loss are presented in panel (B).

increasing. This may explain that the experimentally measured peak frequency is slightly higher than the simulated one.

Note S10. Measured real parts of transmitted sound fields by hybrid metasurfaces

Figure S11 shows the real parts of sound pressure fields of axial focusing and vortex beam focusing. From the 3D full-simulated results in Figure S11(A) and S11(C), it can be seen that sound waves are gathered to the central axial through MS_2 with axial focusing or vortex beam focusing functions. The measured and simulated results in Figure S11(B) and S11(D) show a very good agreement. In addition, compared with sound fields with axial focusing, the unique wave propagation feature with double-lobe alternation can also be clearly observed from Figure S11(D). Figure S12 shows the real parts of sound pressure fields of three types of non-diffraction sound vortex beams. It is observed that the generated three types of vortex beams are less divergent due to the non-diffraction propagation of Bessel beams. The measured real parts of sound pressure fields also show the good consistency to the simulated results.

Note S11. Optimized MS_1 with thinner thickness for water-air sound transmission enhancement

Following the similar optimization procedure, the MS_1 with thinner thickness can be also inversely designed. In this case, the target frequency is chosen at 1715 Hz. The optimized MS_1 has the width and

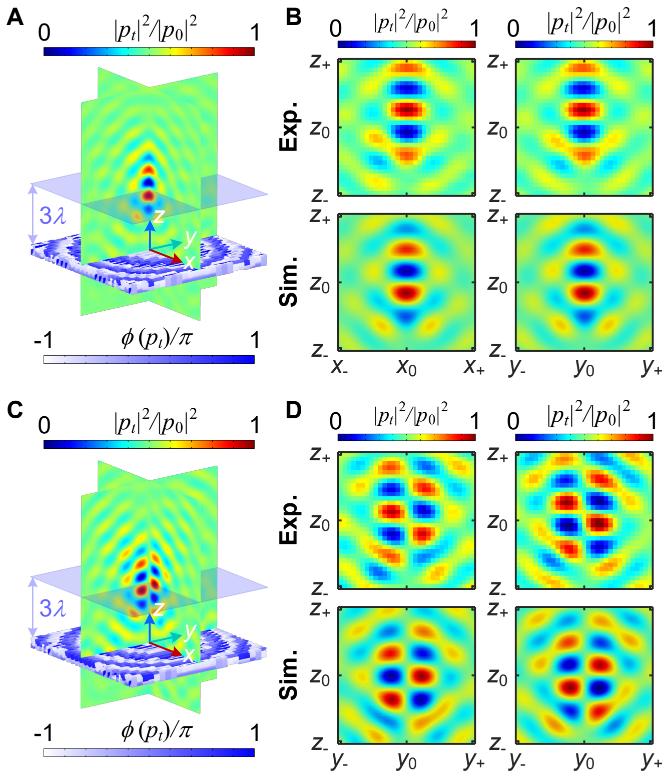

Figure S11: Real parts of sound pressure fields of axial focusing and vortex beams focusing. Panels (A) and (C) show the 3D full-wave simulated real parts fields from air to air of MS_2 for sound axial focusing and vortex beams focusing, respectively. Panels (B) and (D) present the measured real parts of sound fields from water to air in $x - z$ and $y - z$ planes through hybrid metasurfaces at 11 kHz for axial focusing and vortex beams focusing, respectively. The corresponding simulated results obtained from panels (A) and (C) are also given below for comparison. The normalized pressure $|p_0|$ is chosen as 4.1 Pa and 2.5 Pa in panels (B) and (D) for experiments, respectively. $z_0 = 3\lambda_a = 93.5$ mm, $x_0 = y_0 = 0$ mm, $z_\pm = z_0 \pm 60$ mm, $x_\pm = x_0 \pm 60$ mm and $y_\pm = y_0 \pm 60$ mm.

thickness of $\lambda_a/6$ and $\lambda_a/10$ and is divided into 40×24 pixels, respectively. Figure S13(A) presents the water-air sound pressure transmission of optimized MS_1 without and with losses as a function of frequency and the optimized topological configuration. Water-air sound transmission of 99.63% and 76.85% are observed at the peak frequency for lossless and lossy cases, respectively. Meanwhile, the optimized MS_1 also exhibits more than 20 dB transmission enhancement over a relatively wide frequency range from 1445 Hz to 1895 Hz, as marked by the shadow area in the lossless case. Figure S13(B) plots the variations of water-to-air sound transmission through MS_1 and the bare water-air interface with the incident angle θ_w at the peak frequency 1715 Hz. It is observed that the MS_1 can improve the sound pressure transmission over 91.05% within $60°$ oblique incidence, with a contrast to around 3.3% transmission by the bare water-air interface. Furthermore, the full-wave simulated sound pressure fields

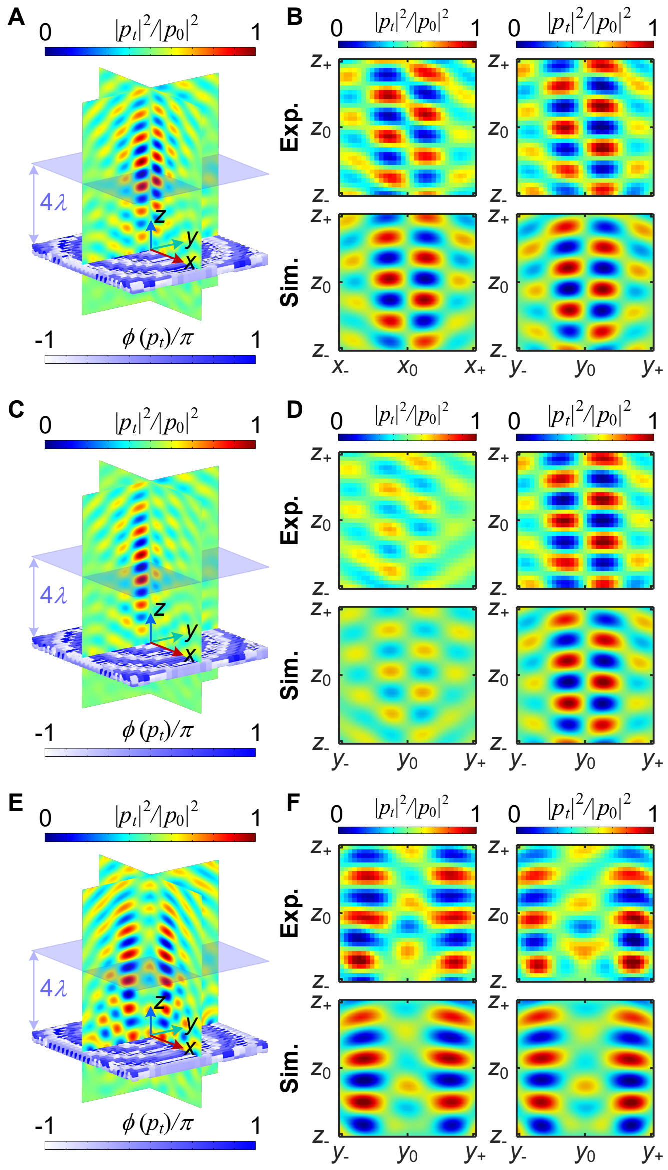

Figure S12: Real parts of sound pressure fields of various non-diffracting sound vortex beams. Panels (A), (C) and (E) show the 3D full-wave simulated real parts fields from air to air of MS_2 for generating the three types of non-diffracting sound vortex beams, respectively, respectively. Panels (B), (D) and (F) present the measured real parts of sound fields from water to air in $x-z$ and $y-z$ planes through hybrid metasurfaces at 11 kHz for the three types of vortex beams, respectively. The corresponding simulated results obtained from panels (A), (C) and (E) are also given below for comparison. The normalized pressure $|p_0|$ is chosen as 1.8 Pa, 2.2 Pa and 1.08 Pa in panels (B), (D) and (F) for experiments, respectively. $z_0 = 4\lambda_a = 124.7$ mm, $x_0 = y_0 = 0$ mm, $z_\pm = z_0 \pm 60$ mm, $x_\pm = x_0 \pm 60$ mm and $y_\pm = y_0 \pm 60$ mm.

through MS_1 and the bare water-air interface are presented in Figure S8(C) and S8(D), respectively. Under single-angle oblique incidence and wide-angle point source incidences, the optimized MS_1 with thinner thickness also exhibits wide-angle sound transmission enhancement across water-air interface.

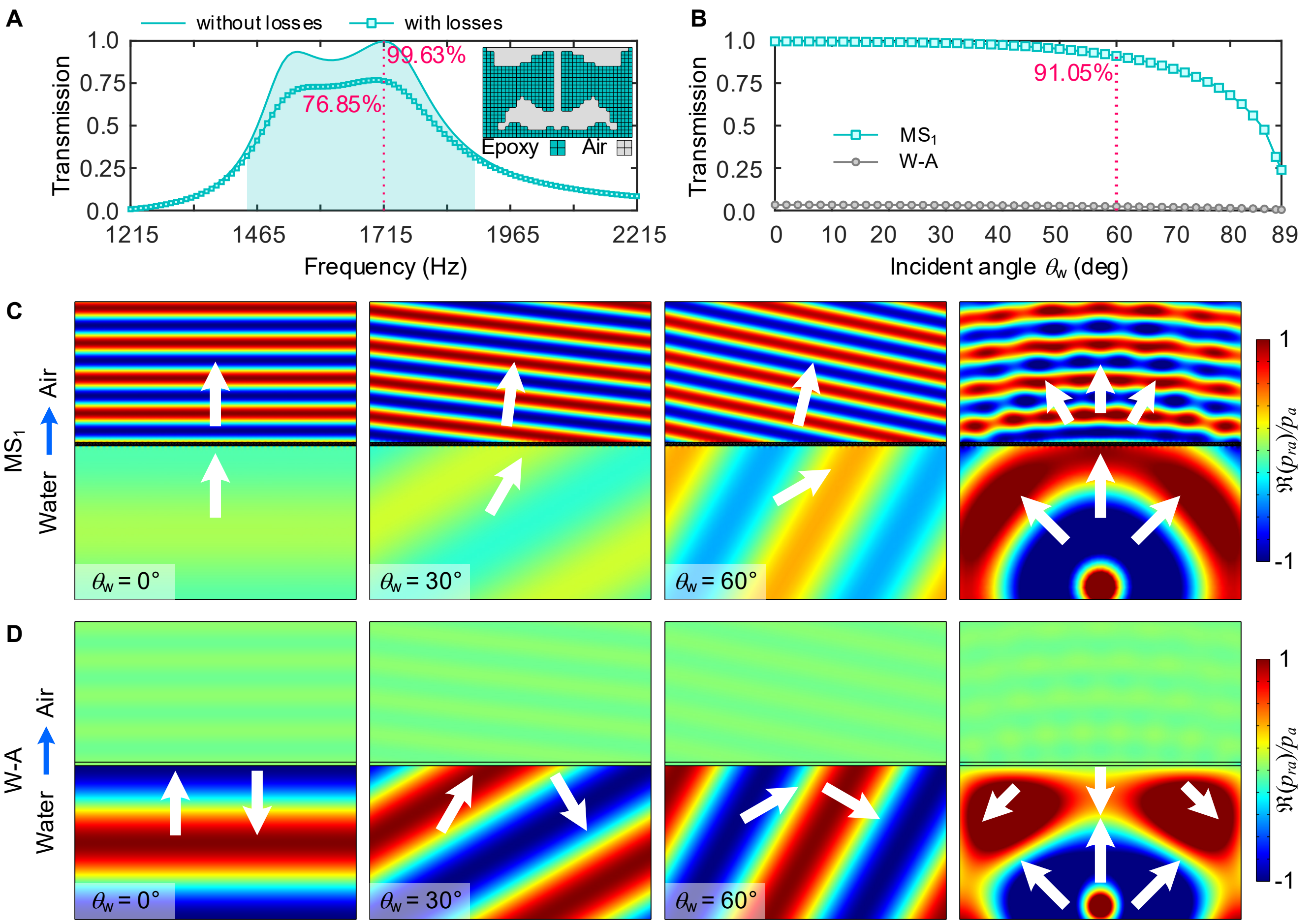

Figure S13: Optimized MS_1 with thinner thickness ($\lambda_a/10$) for water-air sound transmission enhancement. Panel (A) presents the water-to-air sound pressure transmission of MS_1 without and with losses as a function of frequency, where the topological configuration of optimized MS_1 is shown in the inset. The shadow area indicates the frequency range with over 20 dB amplitude enhancement of MS_1 than the bare water-air interface. The variation of water-to-air sound pressure transmission through MS_1 with the incident angle at the peak frequency 1715 Hz is plotted in panel (B). Panels (C) shows the simulated reflected and transmitted sound pressure fields through MS_1 under different incident angles and a point source. The corresponding results through a bare wtaer-to-air interface are presented in panel (D) for comparison. The normalized pressure $p_a = \sqrt{Z_w \cos\theta_a}$ for the water domain and $p_a = \sqrt{Z_a \cos\theta_w}$ for the air domain, where θ_a being the transmission angle in air and θ_w satisfy the Snell's law $k_a \sin\theta_a = k_w \sin\theta_w$.

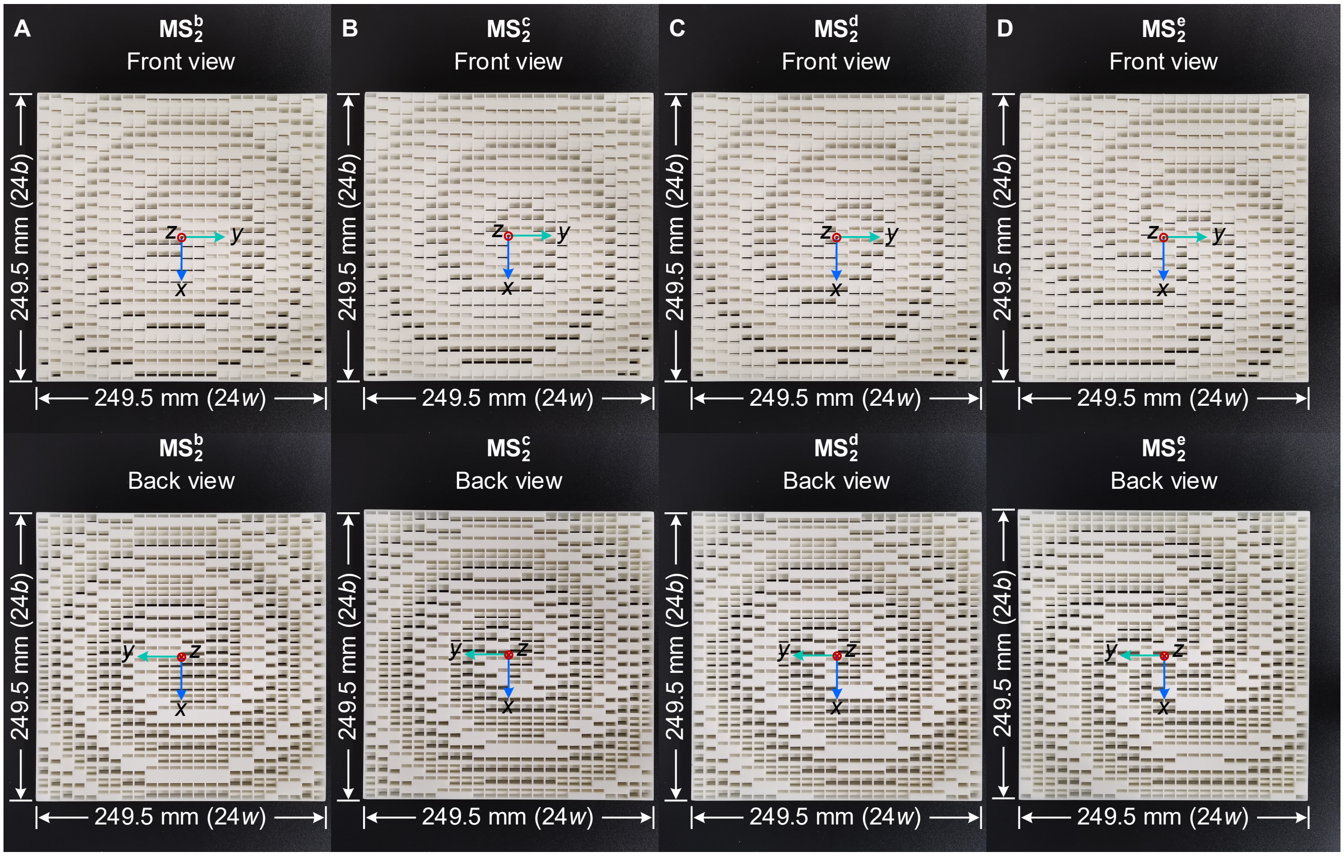

Figure S14: Panels (A)-(D) show enlarged front and back views of fabricated MS_2^b, MS_2^c, MS_2^d, and MS_2^e samples, respectively.

Movie S1.

Supplementary Movie S1 shows the enhanced water-air sound transmission under broadband pulse incidence from 8 kHz to 16 kHz through MS_1 in the experiment. Enhanced water-to-air sound transmission in the wide frequency range is experimentally observed. In addition, at the peak frequency 10.45 kHz, sound transmission amplitude through the bare water-air interface is about 1.2 mPa (35.6 dB). However, the measured sound pressure amplitude in air increases to about 27.8 mPa (62.8 dB) when MS_1 is placed on the water-air interface.

Movie S2.

Supplementary Movie S2 shows the enhanced water-air sound transmission under the peak frequency through MS_1. It is experimentally observed that the sound pressure transmission amplitude is enhanced by about 26.5 dB at the peak frequency 10.45 kHz.

Movie S3.

Supplementary Movie S3 shows the performance of the hybrid metasurfaces for enhanced water-air sound axial focusing in the experiment. Nearly 42 dB enhancement in sound transmission amplitude by hybrid metasurfaces is experimentally observed near the focus at the manipulated frequency 11 kHz.

Movie S4.

Supplementary Movie S4 shows the contrast between experimental and simulated vibration modes of MS_1 at different frequencies. A good agreement is observed between both.
